# Targeted insertion of large DNA sequences by homology‐directed repair or non‐homologous end joining in engineered tobacco BY‐2 cells using designed zinc finger nucleases

**DOI:** 10.1002/pld3.153

**Published:** 2019-07-19

**Authors:** Andreas Schiermeyer, Katja Schneider, Janina Kirchhoff, Thomas Schmelter, Natalie Koch, Ke Jiang, Denise Herwartz, Ryan Blue, Pradeep Marri, Pon Samuel, David R. Corbin, Steven R. Webb, Delkin O. Gonzalez, Otto Folkerts, Rainer Fischer, Helga Schinkel, W. Michael Ainley, Stefan Schillberg

**Affiliations:** ^1^ Fraunhofer Institute for Molecular Biology and Applied Ecology IME Aachen Germany; ^2^ Corteva Agriscience Indianapolis IN USA; ^3^ Indiana Biosciences Research Institute Indianapolis IN USA

**Keywords:** DNA recombination, electroporation, gene targeting, genome editing, particle bombardment, split marker genes, zinc finger nucleases

## Abstract

Targeted integration of recombinant DNA fragments into plant genomes by DNA double‐strand break (DSB) repair mechanisms has become a powerful tool for precision engineering of crops. However, many targeting platforms require the screening of many transgenic events to identify a low number of targeted events among many more random insertion events. We developed an engineered transgene integration platform (ETIP) that uses incomplete marker genes at the insertion site to enable rapid phenotypic screening and recovery of targeted events upon functional reconstitution of the marker genes. The two marker genes, encoding neomycin phosphotransferase II (*nptII*) and *Discosoma sp*. red fluorescent protein (*DsRed*) enable event selection on kanamycin‐containing selective medium and subsequent screening for red fluorescent clones. The ETIP design allows targeted integration of donor DNA molecules either by homology‐directed repair (HDR) or non‐homologous end joining (NHEJ)‐mediated mechanisms. Targeted donor DNA integration is facilitated by zinc finger nucleases (ZFN). The ETIP cassette was introduced into *Nicotiana tabacum* BY‐2 suspension cells to generate target cell lines containing a single copy locus of the transgene construct. The utility of the ETIP platform has been demonstrated by targeting DNA constructs containing up to 25‐kb payload. The success rate for clean targeted DNA integration was up to 21% for HDR and up to 41% for NHEJ based on the total number of calli analyzed by next‐generation sequencing (NGS). The rapid generation of targeted events with large DNA constructs expands the utility of the nuclease‐mediated gene addition platform both for academia and the commercial sector.

**Abbreviations**
4CL5: 4‐coumarate:CoA ligase 5
AHAS: acetohydroxyacid synthase
CRISPR: clustered regularly interspaced short palindromic repeats
Cas9: CRISPR‐associated protein 9
DSB: double‐strand break
*DsRed*: *Discosoma sp*. red fluorescent protein
EC: event characterization
ETIP: engineered transgene integration platform
gDNA: genomic DNA
GFP: green fluorescent protein
GOI: gene of interest
HDR: homology‐directed repair
indels: insertions or deletions
MS: Murashige‐Skoog
NGS: next‐generation sequencing
NHEJ: non‐homologous end joining
nos: nopaline synthase
*nptII*: neomycin phosphotransferase II
PAL: phenylalanine ammonium lyase
PE: paired‐end
TALEN: transcription activator‐like effector nuclease
TCL: target cell line
TR: thioreductase‐like protein
ZFN: zinc finger nuclease

## 1 INTRODUCTION

With the development of programmable, sequence‐specific endonucleases, targeted manipulation of higher plant genomes became a practical reality (Puchta & Fauser, 2014; Zhu et al., 2017) . The introduction of DNA double‐strand breaks by these nucleases triggers the endogenous DNA repair machinery that seals these DSB either by homology‐directed repair or non‐homologous end joining, also known as illegitimate recombination (Waterworth, Drury, Bray, & West, 2011). The HDR mechanism uses DNA with regions homologous to the sequence around the lesion as template for precise repair of the defect. In contrast, NHEJ‐mediated repair is independent of homologous DNA sequences but is more error‐prone, as insertions or deletions (indels) can occur at the DSB site (Gorbunova & Levy, 1997). Both repair mechanisms can be exploited for genome engineering to delete, modify, or add gene sequences of interest at preselected sites in the genome (Voytas, 2013).

Several different nuclease designs have been harnessed to achieve targeted genome modifications in plants (Da Ines & White, 2013). Among them are meganucleases, or homing endonucleases (Stoddard, 2011), zinc finger nucleases (Lloyd, Plaisier, Carroll, & Drews, 2005; Jiang et al., 2013; Petolino et al., 2010), TALENs (Chen & Gao, 2013), and CRISPR‐Cas9 (Bortesi & Fischer, 2015). While the first three systems rely on protein engineering to achieve sequence‐specific DNA binding, the CRISPR‐Cas9 system exploits RNA–DNA base pairing to make the nuclease home in on the genome target site.

All aforementioned systems have been applied successfully in a range of model plant species and crops for genome engineering. By delivery of sequence‐specific nucleases alone, targeted gene inactivation can be achieved by the induction of indels at the target site, leading to disruptive mutations in the targeted coding region as demonstrated for the *ABI4* gene in *Arabidopsis thaliana* (Osakabe, Osakabe, & Toki, 2010) and the *FAD2* gene family in soybean (Haun et al., 2014), among other examples. The co‐delivery of programmable nucleases together with appropriate donor DNA molecules has been used to edit endogenous loci as demonstrated for the rice *ALS* gene to engineer herbicide‐tolerant plants (Sun et al., 2016) or to add genes as demonstrated for the targeted integration of transgenic herbicide resistance markers in corn (Ainley et al., 2013) and cotton (D'Halluin et al., 2013).

Although targeted gene addition has proven effective, selection and characterization of gene targeting events is a cumbersome and time‐consuming process. To address the difficulties associated with the identification and characterization of targeted events, the present study describes the development of a versatile platform for the rapid recognition of targeted events. The design facilitates targeted DNA integration with up to 20‐kb payload either by HDR‐ or NHEJ‐mediated mechanisms.

## 2 EXPERIMENTAL PROCEDURES

### 2.1 ETIP and construct design

**Figure 1:**
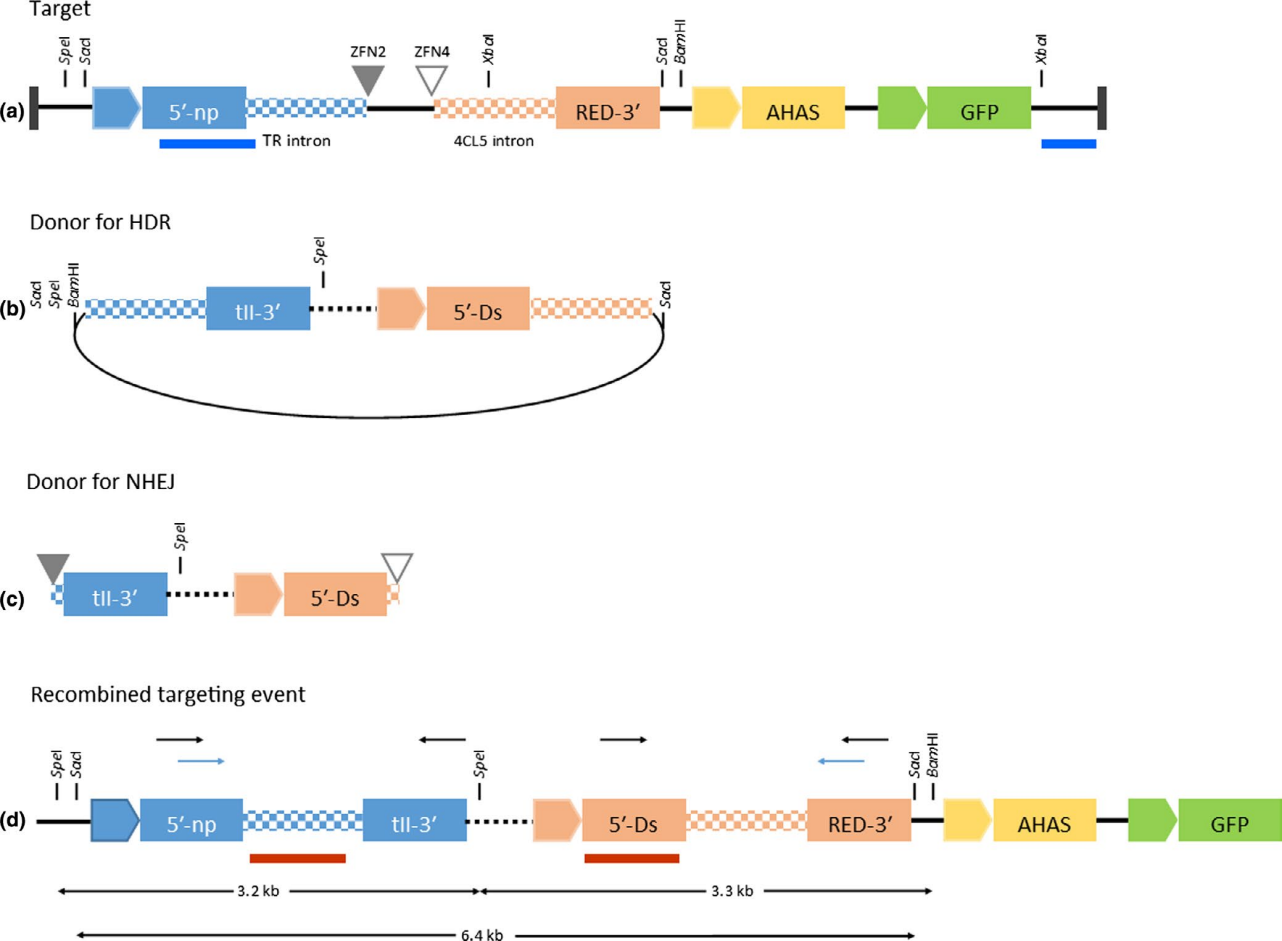
Schematic representation of the ETIP concept. The target construct (a) consists of the 5′ part of the *npt* II gene driven by the nos promoter flanked by the TR intron, the 3′ part of the *DsRed* coding region preceded by the 4CL5 intron, the AHAS gene driven by the Arabidopsis Ubi3 promoter (Norris, Meyer, & Callis, 1993), and the GFP gene driven by the melon actin promoter (Clendennen, Kellogg, Phan, Mathews, & Webb, 2003). The filled triangle indicates the ZFN2 binding site, and the open triangle indicates the ZFN4 binding site. The region between the ZFN sites is a spacer of 131 bp. Probes that were used for checking single copy insertion in target cell line establishment are indicated in blue. The donor DNA (b, c) delivers the 3′ part of the *npt* II coding region and the 5′ part of the *DsRed* gene including the enhanced 35S promoter. Donor DNA contains either flanking homology arms to facilitate HDR‐mediated integration (b) or intron ends providing donor or acceptor sequences flanked by ZFN recognition sites (c) to facilitate NHEJ‐mediated integration. The dashed line indicates the region that accommodates additional payload DNA. The transgenic locus with reconstituted marker genes after successful targeted integration of the donor DNA is shown in (d). Probes that were used for targeted donor DNA insertion are indicated as red bars. Sizes of DNA fragments released upon *Spe*I/*Bam* HI double digestion or *Sac*I digestion are indicated. Primer pairs used for junction PCR are indicated as black arrows, and primers used to detect unmodified target DNA are indicated as blue arrows. Restriction enzyme sites that have been used for Southern blot analyses are shown

The target construct on pDAB113628 (Figure 1a; sequence in Figure S6) contains the two partial marker genes, *npt*II encoding aminoglycoside‐3′‐phosphotransferase II conferring kanamycin resistance (Fuchs et al., 1993) and the *DsRed* gene from *Discosoma* sp., coding for a red fluorescent protein (Jach, Binot, Frings, Luxa, & Schell, 2001). The partial genes were derived from genes internally interrupted (split) by inserting intron sequences and creating splice donor and splice acceptor sites. The *npt*II cDNA was split between nucleotide positions 613–614 within the coding sequence for the substrate recognition domain of the enzyme (Nurizzo et al., 2003). The *DsRed* gene was split between nucleotide positions 209–210 immediately 3′ of the sequence encoding the fluorophore of the mature protein (Yarbrough, Wachter, Kallio, Matz, & Remington, 2001). The 5′ *npt*II part is flanked upstream by the nopaline synthase promoter (Depicker, Stachel, Dhaese, Zambryski, & Goodman, 1982) and downstream by the intron of the Arabidopsis thioreductase‐like protein gene, At3g25580, and the ZFN2 binding site (Ainley et al., 2013). The *DsRed* 3′ part is flanked upstream by the ZFN4 binding site and the intron of the Arabidopsis 4CL5 gene, At3g21230, and downstream by the 35S terminator from the *Cauliflower mosaic virus* (Guilley, Dudley, Jonard, Balazs, & Richards, 1982). For selection of BY‐2 events transformed with pDAB113628, the T‐DNA further contains the cotton AHAS gene (Rajasekaran, Grula, & Anderson, 1996) conferring resistance to the herbicide imazethapyr (Grula, Hudspeth, Hobbs, & Anderson, 1995) and the *TurboGFP* gene derived from *Pontellina plumata* (Evrogen) coding for green fluorescent protein to facilitate the identification of suitable BY‐2 target cell lines.

The donor vectors contain the 3′ part of the *npt*II gene with the nopaline synthase terminator and the 5′ part of the *DsRed* gene with the enhanced 35S promoter (Kay, Chan, Dayly, & McPherson, 1987) to reconstitute the functional *npt*II and *DsRed* genes, respectively, upon integration of donor sequences into pDAB113628 transgenic events. Vector pDAB113676 (sequence in Figure S6), designed for HDR‐mediated integration, contains the full‐length 823 bp TR intron at the 5′ end and 942 bp of the 4CL5 intron at the 3′ end. The NHEJ donor vector, pBlueSKM_NHEJ_ZFN2_ZFN4 (sequence in Figure S6), contains the ZFN2 recognition site followed by 110 bp of the TR intron at the 5′ end and 140 bp of the 4CL5 intron followed by the ZFN4 recognition site.

Large donor vectors were created by inserting non‐coding DNA stretches of 5 kb, 10 kb, 15 kb, or 20 kb between the two marker gene parts.

### 2.2 Plant material and culture conditions

*Nicotiana tabacum* L. cv. BY‐2 suspension cells were cultivated in Murashige‐Skoog (MS) basal medium as previously described (Schneider et al., 2016). The cells were subcultured twice weekly by transferring 5% (v/v) of the culture into 50 ml fresh medium. Working cultures were inoculated with 2%–10% (v/v) of 3‐day‐old cultures and used 3–4 days after inoculation for transformation via particle bombardment and Agrobacterium, respectively, and up to 7 days after inoculation for electroporation. Callus material from transformation events was used to establish suspension cultures in 50‐ml TubeSpin Bioreactors (TPP Techno Plastic Products AG) containing 10 ml selective MS medium (1.5 μM imazethapyr or 100 mg/L kanamycin). After initial subculturing in the bioreactor tubes, cultures were transferred to 50‐ml Erlenmeyer flasks and subcultured once weekly using an inoculum of 3%–5% (v/v).

### 2.3 Agrobacterium‐mediated generation of target cell lines

Target vector pDAB113628 has been introduced into *A. tumefaciens* strain LBA4404 (Invitrogen) by electroporation (Dower, Miller, & Ragsdale, 1988). Transgenic BY‐2 cells were generated by co‐cultivation of Agrobacterium and BY‐2 wild‐type cells as described (An, 1985). Transgenic events were selected on MS agar plates supplemented with 1.5 μM imazethapyr (Sigma Aldrich).

### 2.4 Particle bombardment and electroporation of BY‐2 cells

Biolistic DNA delivery was carried out using the Biolistic PDS‐1000/He^TM^ Particle Delivery System as described previously (Schneider et al., 2016). In brief, BY‐2 cell aliquots of 600 μl packed cell volume from working cultures were incubated on filters on solid MS medium or MS medium supplemented with osmotica (32 mM mannitol and 32 mM sorbitol) at room temperature approximately 1 hr prior to bombardment. Donor and ZFN2/ZFN4 plasmid DNA at a molar ratio of 4:1 or 4:1:1 were coated onto 0.6 μm diameter gold particles with a total DNA amount of 3–5 μg for 10 shots and precipitated by CaCl_2_ and spermidine. Bombardment was performed with a helium pressure of 650 psi and a flight distance of 9 cm. The filters from MS medium supplemented with osmotica were moved to plates with MS medium directly after bombardment. Cells were kept at room temperature for 2 days to regenerate and distributed to four plates with selective MS medium (100 mg/L kanamycin) for callus formation at 28°C.

Electroporation of BY‐2 protoplasts was done as described before (Schneider et al., 2016). The ratio between donor DNA and ZFN coding DNA was 4:1; the ratio of ZFN2:ZFN4 was 1:1, 1:2, or 1:4.

### 2.5 qPCR

Genomic DNA was extracted from 100 mg of callus material using the chemagic DNA plant kit (PerkinElmer chemagen) according to the manufacturer's instructions. Primer pairs for gene‐specific amplification of the Pnos promotor (Pnos_for, Pnos_rev), TurboGFP (tGFP_for2, tGFP_rev2), and PAL gene (Nt_PAL_for, Nt_PAL_rev) (Fukasawa‐Akada, Kung, & Watson, 1996) were manually designed using Clone Manager software according to primer standards (sequences in Table S1) and custom‐ordered from Eurofins Genomics. Three replicates of each genomic DNA sample were analyzed by amplification of the target sequences on an ABI Prism 7500 Sequence Detection System (Applied Biosystems) using the Platinum^®^ SYBR^®^ Green SuperMix‐UDG with ROX Kit for quantitative real‐time PCR (Invitrogen) with the following parameters: 2 min at 50°C and 10 min at 95°C, followed by 40 cycles of 15 s at 95°C and 45 s at 60°C. Normalization of the mean Ct values of both target genes (Pnos and TurboGFP) to the mean Ct values of the PAL reference gene and the calculation of the relative gene copy numbers were performed according to the 2^−ΔΔCt^ method (Livak & Schmittgen, 2001).

### 2.6 Junction PCR for identification of targeted integration events

The integration of the donor DNA into the ETIP cassette was evaluated using a PCR approach with primer sets npt5_F5 and nptII_3′ UTR_rev2 for the 5′ border and rfp5_F and rfp3 for the 3′ border. When donor backbone was integrated on the *DsRed* side, primer sets dbbr_F2 and rfp3 or dbbr_F2 and RFP3_R2 were used (primer sequences in Table S1). PCR was carried out using the i‐MAX II polymerase kit (iNtRON biotechnology, Korea) with an annealing step of 20 s at 61°C for 40 cycles using 9 μl (20–60 ng) of the gDNA as template.

### 2.7 PCR for identification of unmodified target DNA

To check for the presence of residual target after successful integration of the donor DNA (i.e., non‐clonal calli), an out–out PCR with primer set disF and disR (Table S1) binding 5′ of the homology region in the TR intron and 3′ of the homology region in the 4CL5 intron. PCR was carried out using the i‐MAX II polymerase kit with an annealing step of 15 s at 61°C for 35 cycles using 9 μl (20–60 ng) of the gDNA as template.

### 2.8 DNA sequencing (Sanger)

PCR products generated by junction PCR using the primer sets mentioned above were sequenced to verify a seamless and correct integration of the donor into the ETIP cassette. Sequencing on an ABI PRISM 3730 Genetic Analyzer was carried out in separate reactions using primer rfp3 and rfp5_F for *DsRed* as well as npt5_F5 and nptII_3′ UTR_rev2 for *npt*II to sequence the respective reconstituted gene (primer sequences in Table S1).

### 2.9 Next‐generation sequencing (NGS)

To identify intact single copy targeted integrations of donor DNAs at the expected ETIP target site in the tobacco genome, a sequence capture‐based NGS event characterization (EC) procedure (Guttikonda et al., 2016) was employed. Genomic DNA from selected samples was extracted and sheared by sonication to ~800 bp fragments. Sheared DNA was hybridized to a collection of 120 bp overlapping complementary probes specifically designed for target, donor, and ZFN construct DNA sequences used in this study. The resulting DNA libraries were sequenced to produce 300‐bp paired‐end (PE) reads on an Illumina MiSeq sequencer in 48‐plex pools.

First, samples containing ETIP integrations only were subjected to the standard EC computational analysis procedure to confirm successful integrations of ETIP DNA fragment in TCL lines (Guttikonda et al., 2016). For targeted events, in addition to the standard analysis, modifications to the standard pipeline were made to accommodate the unique situations for targeted genome editing in tobacco. As the tobacco genome assembly was split into 420,000 scaffolds, an iterative approach was used to characterize transgene integration.

To reduce ambiguity, homologous regions were removed from both donor and ETIP sequences to generate modified genome references to identify unambiguous PE read pairs spanning the donor/ETIP junctions. For NHEJ‐mediated events, individual read analysis was conducted to characterize the ZFN cleavage. In addition, a whole‐genome level analysis was done to check for any evidence of integration of ZFN constructs in tobacco genome outside the targeted region.

To detect possible mutations introduced in the integration process, standard variant calling process for short reads was incorporated as part of the EC pipeline. For each position in the donor, if sequencing coverage is larger than or equal to 100, and over 95% of the reads support an alternative allele, we record the position as a variant in the SNP summary table. The standard EC pipeline is available through Guttikonda et al. (2016).

### 2.10 Flow cytometry analysis

Protoplasts from BY‐2 suspension cultures were isolated as described (Schinkel, Jacobs, Schillberg, & Wehner, 2008). Qualitative analysis of the TurboGFP fluorescent cell population was investigated using FACSVerse flow cytometer (BD Bioscience). The viable cell population was gated based on light scatter signals (SSC‐A and FSC‐H) while the percentage of green fluorescent protoplasts in each culture was detected at 527/32 nm (FITC‐H; GFP). Two types of protoplasts were used to set the gates for the presence of green fluorescence: wild‐type BY‐2 protoplasts and protoplasts derived from TurboGFP expressing cell line C#86 (Schneider et al., 2016). We analyzed 10^4^ viable gated protoplasts for each transgenic culture and processed the signal data with the FACSuite Software (BD Bioscience).

### 2.11 Southern blot analysis

Genomic DNA prepared from suspension‐cultured cells was digested with *Xba*I, *Bam*HI/*Spe*I, or *Sac*I (NEB) and separated on a 0.6% (small donors) or 0.4% (large donors) (w/v) agarose gel at 60 V for 3 hr. Prior to transfer, the DNA was depurinated by incubating the gels in 0.25 M HCl for 15 min. The DNA was subsequently denatured by incubation in 0.5 M NaOH and 0.5 M NaOH/1.5 M NaCl for 30 min each. After neutralization (1 M Tris, 1.5 M NaCl, pH 7.0) for 30 min, the DNA was transferred to a positively charged nylon membrane (Carl Roth) by vacuum transfer with the Vacu‐Blot device according to the manufacturer's instructions (Biometra, Göttingen, Germany) using 2 × SSC. The DNA was immobilized on the membrane by incubation at 80°C for 2 hr. Probes were labeled using α^32^P‐dATP (Hartmann Analytic) and the DecaLabel DNA labeling kit (Thermo Fisher Scientific). Hybridization was performed using the Roti‐Hybri‐Quick solution (Carl Roth) according to the manufacturer's instructions. For probe preparation, the following regions were PCR amplified from pDAB113628: 48‐1041 (3′ end of target T‐DNA), 8455‐9491 (5′ end of target T‐DNA), and 9044‐9784 (TR intron). Region 2181‐2891 was PCR amplified from pDAB113676 to prepare the *DsRed*_5′probe.

## 3 RESULTS

### 3.1 Design of the engineered transgene integration platform (ETIP)

To establish a gene targeting platform that enables convenient phenotypic identification of targeted events, DNA constructs were designed that comprised a selectable and a screenable marker gene into which introns were added, thus, allowing each gene to be easily split in half between the target and the donor DNA constructs (Figure 1). The marker genes are *npt*II, encoding aminoglycoside‐3′‐phosphotransferase II conferring kanamycin resistance (Fuchs et al., 1993), and the *DsRed* gene from *Discosoma* sp. coding for a red fluorescent protein (Jach et al., 2001) to enable visual screening of targeted events. The donor DNAs (Figure 1b,c) deliver the missing 3′ end of the *npt*II gene, including its terminator, and the missing 5′ end of the *DsRed* gene, including its promoter. After a successful targeting event (Figure 1d), the 5′ and 3′ ends of the marker genes are joined via intron sequences derived from a thioreductase‐like protein (TR) gene in case of *npt*II or a 4‐coumarate:CoA ligase gene (4CL5) for *DsRed*. The individual marker gene parts were shown to be nonfunctional; the intron‐containing genes confer kanamycin resistance or red fluorescence as expected (Figure S1).

To facilitate the identification of a suitable BY‐2 target cell line, the T‐DNA of the target construct contained the cotton acetohydroxyacid synthase (AHAS) gene cassette conferring resistance to the herbicide imazethapyr (Grula et al., 1995) and the GFP gene from *Pontellina plumata* coding for a green fluorescent protein.

### 3.2 Establishment of BY‐2 target cell lines

Transgenic tobacco BY‐2 cells carrying the ETIP cassette were generated by Agrobacterium‐mediated transformation, and the final target suspension cell lines (TCL) were selected in the process detailed in Figure S2a. A total of 958 imazethapyr‐resistant transformants were recovered. To analyze the integrity of the ETIP cassette and estimate its copy number, a real‐time PCR analysis was performed on genomic DNAs (gDNAs) from the transformants with two primer pairs binding to either the 5′ or 3′ end of the ETIP cassette. The intron of the phenylalanine ammonia‐lyase (PAL) gene was used as an endogenous reference. Based on the analysis, 108 lines with low target copy numbers were chosen for further characterization by Southern blot analysis. To this end, genomic DNA was digested with *Xba*I and hybridized with a DNA probe binding to the 3′ end of the ETIP cassette close to the left border of the T‐DNA (scheme in Figure 1a). This analysis showed a single insertion of the ETIP cassette for 70 of 108 analyzed lines. On a representative Southern blot (Figure S2b), five lines out of ten had a single band (TCL#157, #218, #340, #403, and #448). In order to validate that the integration locus supports sustained gene expression, all 70 lines were used to produce protoplasts that were analyzed by flow cytometry to determine the fluorescence intensity of GFP (Figure S2c). Lines were evaluated based on the intensity of the green fluorescence signal in comparison with the previously described BY‐2 cell line C#86 containing the same GFP cassette (Schneider et al., 2016). Fourteen clones with a relatively high GFP fluorescence (≥fluorescence of C#86) were kept as candidates for the establishment of TCL. Single copy integration of the ETIP cassette in these 14 lines was reappraised with a second probe binding close to the right border to the 5′ part of the *npt*II gene by probing *Xba*I‐digested gDNA on a Southern blot (Figure S3). Based on this analysis, six TCLs, which showed signals indicating a second integration of the ETIP cassette or part thereof, were excluded from further work. As it was intended to develop the TCL as a platform for the targeted integration of diverse genes of interest (GOI) in various projects, the remaining 8 lines were further evaluated for the stability of GFP expression over time (12 weekly subcultures) to ensure the target construct has been inserted in a locus that supports sustained gene expression (Figure S4). Three TCLs did not display a stable GFP production and were excluded from further analysis. For subsequent targeting experiments, TCL#448 was used as the target cell line. In this cell line, the ETIP has been integrated in an intergenic region leaving a minimal deletion footprint of 22 bp as determined by NGS. Using a BLAST search against the tobacco genome sequences deposited at the SOL genomics network (Fernandez‐Pozo et al., 2015), we retrieved the scaffold Ntab‐BX_AWOK‐SS2405 containing the sequence corresponding to the ETIP integration site (position 33663–33684) (Figure S2d).

### 3.3 Gene targeting by HDR‐mediated integration

**Figure 2:**
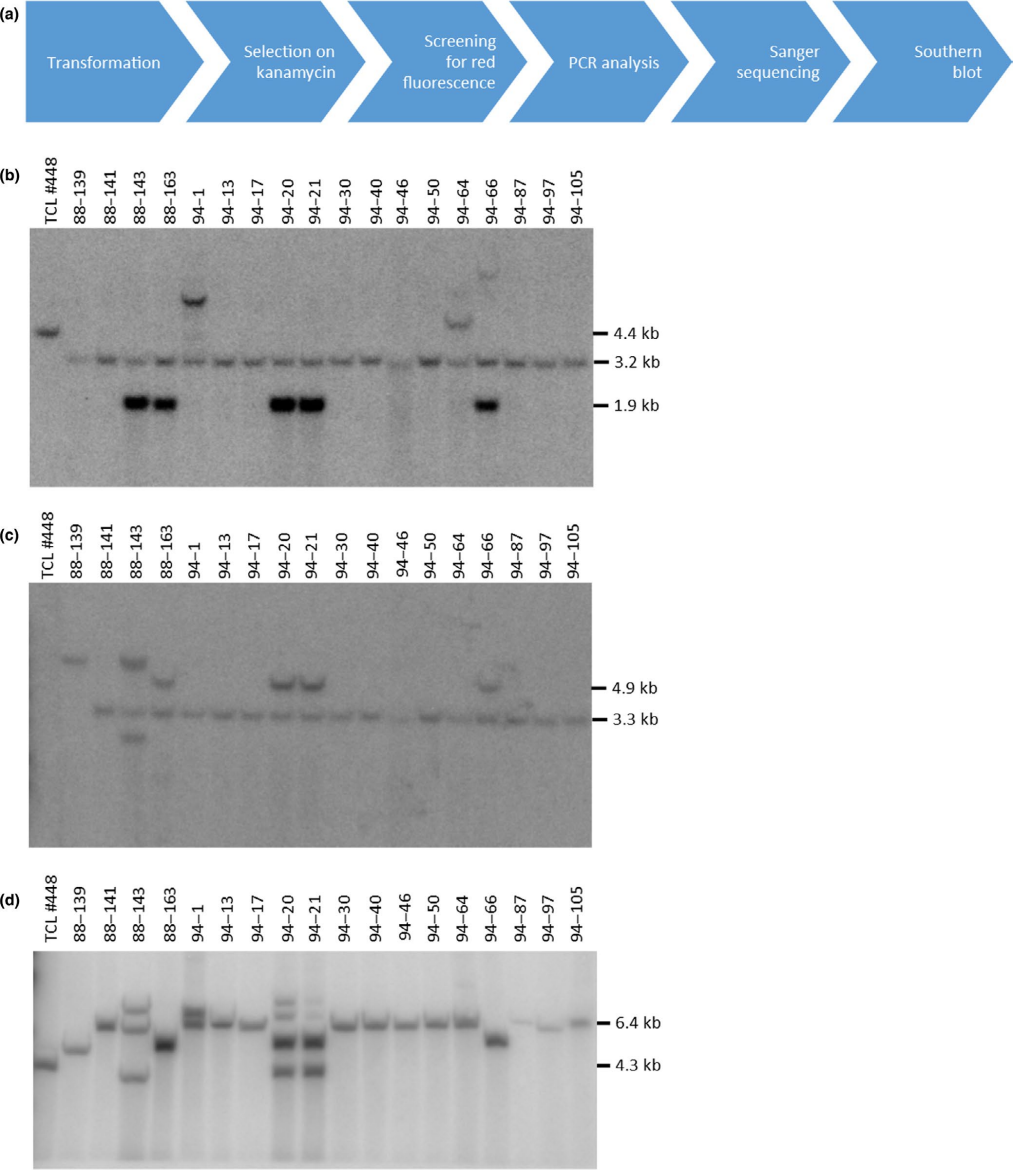
HDR‐mediated targeted gene addition in TCL#448. (a) The workflow for event generation and screening. Eighteen events were finally selected for a variety of Southern blot analyses. (b) gDNA was digested with *Bam* HI/*Spe*I and the resulting fragments hybridized with a TR intron probe. Fragment sizes for unmodified target DNA (4.4 kb), targeted integration (3.2 kb), and random integration (1.9 kb) are indicated. (c) The same Southern blot as in (b) was hybridized with a probe binding to the 5′ part of the *DsRed* gene. Given fragment sizes indicate targeted (3.3 kb) or random integration (4.9 kb). (d) gDNA was digested with *Sac*I, cutting both restored markers from the genomic DNA, which is shown by hybridization with the TR probe as used in (b). Given fragment sizes indicate targeted integration (6.4 kb) and unmodified target DNA (4.3 kb)

To further characterize functionality of the TCL for targeting, HDR‐mediated gene targeting was performed by co‐bombarding TCL#448 cells with donor DNA vector pDAB113676 containing the donor as described above (Figure 1b) as well as ZFN2 and ZFN4 coding plasmids pDAB105962 and pDAB105964. Candidate events for targeted donor DNA integration were isolated and analyzed as outlined in Figure 2a and Table 1. Transformed cells were selected on kanamycin‐containing agar plates, and kanamycin‐resistant calli were screened for red fluorescence (Figure S5a). A subset of positive clones was selected for molecular analysis. Genomic DNA was extracted and used for junction PCRs on both borders and for target‐specific PCR to prove the empty landing pad was not present. Out of 41 randomly selected clones that were kanamycin‐resistant and showed red fluorescence, 30 clones passed all PCR tests, 27 of which underwent sequencing of the generated junction PCR products and showed all correct sequences (Table 1). Finally, a subset of 18 clones was subjected to Southern blot analysis to verify the PCR data and determine whether randomly integrated donor DNA copies are present. By hybridization with a TR‐intron‐specific probe (Figure 2b), the recombined 3.2‐kb *Spe*I fragment specific for targeted integration of the donor DNA was detected in all samples and seven events had randomly integrated donor DNA copies as shown by the presence of *Bam*HI/*Spe*I fragments of various sizes. The most prominent band migrating at 1.9 kb is derived from a *Bam*HI/*Spe*I fragment of the donor vector comprising the TR intron sequence including the *Bam*HI site from the vector backbone and the *npt*II 3′ part (Figure 1b). Stripping and re‐probing the blot with a probe specific for the 5′‐coding region of *DsRed* (Figure 2c) confirmed successful targeted integration, indicated by a 3.3‐kb hybridizing band, and additional random integration of donor vector DNA, indicated by a 4.9‐kb *Spe*I/*Spe*I fragment comprising the *DsRed* 5′ part, the 4CL5 intron, and vector backbone sequences including a *Spe*I site (Figure 1b). For event 88‐139, the expected 3.3‐kb fragment is not present and instead a 6‐kb fragment appears on the blot consistent with additional integration of donor vector backbone DNA potentially via a NHEJ‐mediated mechanism. In addition, the gDNA samples were digested by *Sac*I to completely release both reconstituted markers from the target locus. In properly targeted events, a 6.4‐kb fragment should be released from the target locus. Eleven of 18 analyzed events (88‐141, 94‐1, 94‐13, 94‐17, 94‐30, 94‐40, 94‐46, 94‐50, 94‐64, 94‐87, and 94‐105) show the expected band demonstrating successful targeted addition of the *npt*II_3′ region and the *DsRed*_5′ region (Figure 2d). Events that showed additional integration of donor DNA copies in the previous Southern blots (88‐143, 88‐163, 94‐20, 94‐21, and 94‐66) did not show the 6.4‐kb band, indicating integration of donor vector backbone DNA (containing additional *Sac*I sites, Figure 1b) at the target locus.

**Table 1:** Analysis of targeted addition of donor DNA (without payload) by HDR‐ or NHEJ‐mediated integration mechanisms

	Kanamycin resistance	Red fluorescence		Junction PCR *DsRed*		Out‐out PCR		Junction PCR *nptII*		*DsRed* border sequences		*nptII* border sequence		Southern blot	
	Clones rescued	Anal.	Positive	Anal.	Positive	Anal.	Positive	Anal.	Positive	Anal.	Positive	Anal.	Positive	Anal.	Positive
HDR biolistics	327	327	36%	41	85%	30	100%	35	86%	27	100%	27	100%	18	94%
NHEJ biolistics	298	298	25%	73	70	48	60%	36	97%	26	100%	21	100%	18	100%
NHEJ electropor	2546	2546	14%	141	80%	99	62%	37	100%	20	100%	20	100%	18	100%

Note

The method of transformation was biolistics for HDR and biolistics or electroporation for NHEJ. Junction PCR for DsRed was performed with primers rfp5_F and rfp3, junction PCR for *nptII* was performed with primers npt5_F5 and nptII_3′UTR_rev2, and out–out PCR was performed with primers disF and disR (for sequences see Table S1). The numbers given under analyzed (Anal.) are the number of calli that were analyzed in that specific step, and the number under positive is the percentage of these calli that were positive. Each subsequent analysis only analyzed part or all of the positive calli of the step before; exception is the out–out PCR and junction PCR for *nptII* for the HDR events, where the junction PCR for *nptII* was performed first and consequently that number of analyzed calli is higher.

### 3.4 Targeted gene insertion by NHEJ

**Figure 3:**
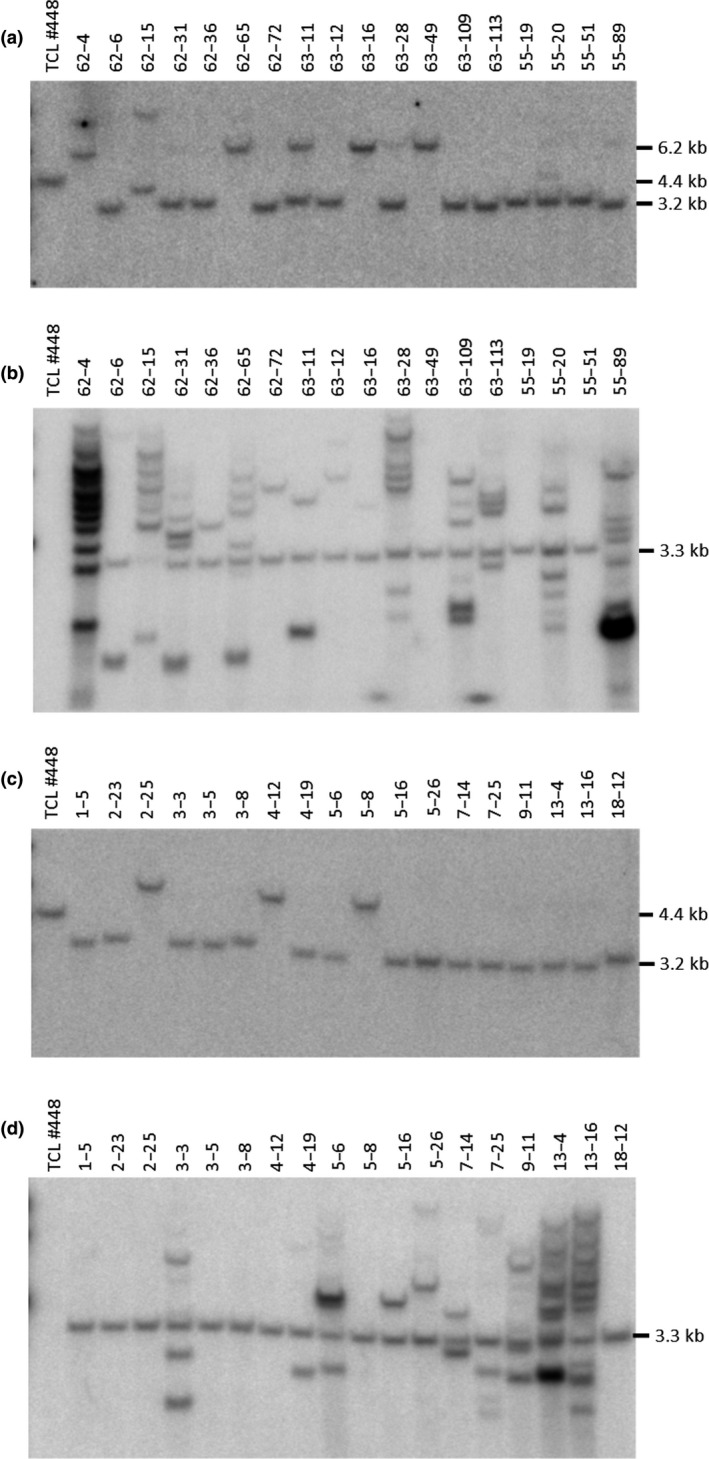
NHEJ‐mediated targeted gene addition in TCL#448. Eighteen events derived from biolistic delivery (a) and (b) or electroporation (c) and (d) of donor and ZFN coding plasmids were subjected to Southern blot analysis. Genomic DNA was digested with *Bam* HI/*Spe*I and the corresponding blots probed using either a TR‐intron‐specific probe (a) and (c) or a *DsRed*_5′ specific probe (b) and (d). A fragment size of 4.4 kb indicates unmodified target DNA, fragment sizes of 3.2 kb (a and c) or 3.3 kb (b and d) indicate targeted integration, and a fragment size of 6.2 kb indicates targeted integration with additional vector backbone DNA

To characterize NHEJ‐mediated integration at the target site, TCL#448 cells were co‐bombarded with a donor DNA encoding the 3′ part of the *npt*II expression cassette and the 5′ part of the *DsRed* expression cassette flanked by short (~100 bp) stretches of the intron sequences that are not present in the target DNA and the ZFN2 and ZFN4 binding sites (Figure 1c). The donor DNA was co‐delivered with plasmids encoding ZFN2 and ZFN4. Seventy‐five of the 298 kanamycin‐resistant events also showed the red fluorescent phenotype (Table 1). The target‐specific and target/donor junction PCR with subsequent sequence analysis identified 21 events that passed all assays. Genomic DNA from 18 events was prepared, digested with *Bam*HI/*Spe*I, and subjected to Southern blot analysis using the TR‐intron‐specific probe (Figure 3a). All 18 events displayed hybridizing bands with sizes different to the original target DNA indicating modification of the target locus consistent with NHEJ‐mediated gene targeting. In 13 of the 18 events, the band showed a size of ~3.2 kb indicating integration at the target site. Slight differences in the mobility of this band in different events suggest the occurrence of repair‐associated indels. For five events (62‐4, 62‐65, 63‐11, 63‐16, and 63‐49), the band migrated with a slower mobility around 6.2 kb consistent with the additional integration of vector backbone DNA. Re‐probing this blot with the probe specific for *DsRed*_5′ coding sequence revealed the expected 3.3‐kb band expected for targeted insertion of the donor DNA. Random integration of donor DNA is apparent in most events, often in multiple copies (Figure 3b). Three of the 18 analyzed events (63‐49, 55‐19, and 55‐51) were free of any detectable randomly integrated donor DNA copies and represent therefore “clean” targeted events.

To evaluate an alternative transformation method for NHEJ‐mediated targeting, donor DNA and ZFN coding vectors were delivered to TCL#448 protoplasts by means of electroporation. Resulting events were screened similarly to the bombardment generated events (Table 1), and 18 selected clones were finally selected for Southern blot analysis as described above. All analyzed events have successfully integrated the donor DNA at the target site as proven by disappearance of the 4.4‐kb target‐specific band. Fifteen events show the 3.2‐kb band diagnostic for integration of the donor DNA (Figure 3c), and three events (2‐25, 4‐12, and 5‐8) have a 6.2‐kb band indicating incorporation of donor vector backbone DNA at the *npt*II side of the target site. The hybridization of the same blot with the *DsRed*_5′ probe (Figure 3d) revealed additional random integration of donor DNA in ten events elsewhere in genome. In total, eight events (1‐5, 2‐23, 2‐25, 3‐5, 3‐8, 4‐12, 5‐8, and 18‐12) are free from random donor integration and are therefore regarded as clean targeted events.

### 3.5 Targeted addition of large donor molecules

A prerequisite for broader application of targeted gene addition for the development of novel crop varieties is the insertion of large DNA molecules for simultaneous integration of multiple traits. The rapidity and robustness of the ETIP system enabled us to generate enough events to explore the feasibility of integrating donor DNAs with increasing payload sizes.

Donor vectors with payloads (essentially random DNA) of 5 kb, 10 kb, 15 kb, or 20 kb were designed. Analogous to the previous donor vectors, these constructs either contained flanking homology arms to facilitate HDR‐mediated integration or just intron sequences flanked by ZFN binding sites for NHEJ‐mediated integration (Figure 1b,c).

All eight donor vectors, four each for HDR‐ and NHEJ‐mediated targeting, were delivered separately to TCL#448 by particle bombardment along with a plasmid encoding ZFN2. In addition, the two donor vectors containing the largest (20 kb) payload were co‐delivered with both ZFN2‐ and ZFN4‐coding plasmids. Targeted events were selected on kanamycin‐containing agar plates. Genomic DNA was prepared from all regenerated calli. Junction PCR analyses were conducted, and all events that were positive for both borders were used to initiate suspension cultures to allow the preparation of larger quantities of high‐quality gDNA. The gDNA samples were used to perform sequence capture‐based NGS to characterize targeted integration of the donor DNA into the ETIP cassette and to identify random integration of donor DNA elsewhere in the genome. The sequence capture NGS data revealed events that contain a complete and targeted donor DNA integration for each of the different size payloads evaluated and each of the two mechanisms employed (Table 2). For HDR‐mediated events, homology regions in donor and ETIP were excluded from the read mapping and sequence assembly process to avoid ambiguity between donor and target.

**Table 2:** Analysis of targeted addition of donor DNA with payload by HDR‐ or NHEJ‐mediated integration mechanisms

	Kanamycin resistance	Red fluorsc.	Junct. PCRs	NGS analysis		
	Calli	Positive		Analyzed calli	Single targeted insertion	Targeted insertion + X
Payload^a^ (HDR)						
5 kb	141	39 (28%)	41 (29%)	39	8 (21%)	18 (46%)
10 kb	126	24 (19%)	36 (29%)	35	1 (3%)	24 (69%)
15 kb	105	11 (11%)	23 (22%)	20	3 (15%)	9 (45%)
20 kb	121	17 (14%)	24 (20%)	18	2 (11%)	8 (44%)
20 kb ZFN2+4	33	6 (18%)	11 (33%)	12	1 (8%)	4 (33%)
Payload^a^ (NHEJ)						
5 kb	90	15 (27%)	41 (46%)	24	5 (21%)	10 (42%)
10 kb	106	22 (21%)	49 (46%)	39	16 (41%)	16 (41%)
15 kb	102	18 (18%)	29 (28%)	26	4 (15%)	15 (58%)
20 kb	80	16 (20%)	9 (11%)	9	1 (11%)	3 (33%)
20 kb ZFN2+4	38	4 (11%)	13 (34%)	16	6 (38%)	7 (44%)

Notes

Junction PCRs were performed with primers npt5_F5 and nptII_3′ UTR_rev2 for *nptII* and primers dbbr_F2 and rfp3 or rfp3_R2 for *DsRed* (see Table S1 for sequences). Success rates are given for each payload/mechanism relative to all events tested for the phenotypical markers and the junction PCRs in number of calli and percentage. For NGS, numbers are given relative to all events analyzed by NGS in calli and percentage.

Abbreviations: fluorsc.: fluorescence; Junct., Junction; X, extra copies of donor DNA integrated either at the target site or at the target site and other non‐target genomic sites.

a Payload is only the exchangeable part of the donor vector; integrated fragments are 2.2 kb larger (marker genes) for HDR and 5.2 kb larger (marker genes and donor vector backbone) for NHEJ.

Frequently, events containing full‐length donor DNA integration at the target site also showed random integrations at non‐target locations in the tobacco genome. Furthermore, multiple donor DNA copies integrated at the ETIP site as a result of tandem concatenation of complete/partial donor copies were found. Correctly targeted single copy events generated by both NHEJ (up to 41% of sequenced events) and HDR (up to 21% of sequenced events) were identified (Table 2). NGS analyses also revealed additional sequence fragmentation and rearrangements that involve integration of reorganized donor DNAs and backbone sequences at the target integration site.

**Figure 4:**
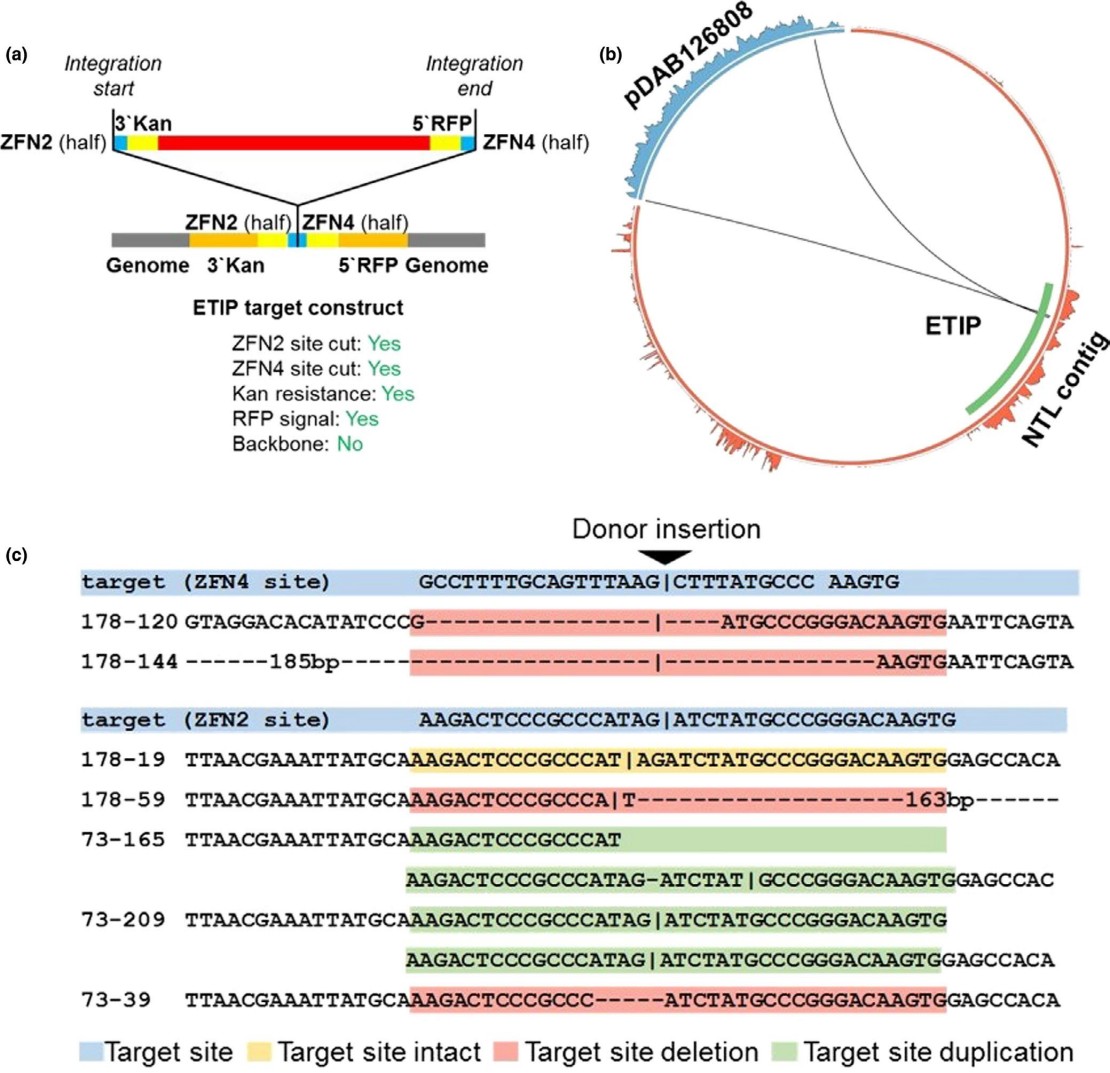
Donor integration at the target site. (a) Diagram of expected ZFN recognition sites (when both ZFN2 and ZFN4 are used), integration into ETIP and reconstitution of marker genes *nptII* and *DsRed* at 5′ and 3′ end, respectively. (b) NGS result of event 178‐144. Both ZFN recognition sites are cut by ZFN proteins, and the donor is integrated into the ETIP target site, reconstituting both marker genes. Blue, donor pDAB126808 and NGS coverage; red, tobacco genome contig Ntab‐BX_AWOK‐SS2405 (here referred as NTLcontig) with ETIP integrated, and NGS coverage; green, ETIP within the tobacco contig. (c) ETIP target site variations as a result of ZFN protein recognition and cutting. Blue, designed target site sequences; yellow, intact target sites; red, target site deletions; green, target site duplications

A focused analysis of NGS data of target donor junctions in NHEJ‐mediated events uncovered target site modifications such as target site deletions and duplications, as well as intact target sites (Figure 4c). The highly variable target site modifications from the integration process suggest that the design of target sites with introns flanking the marker gene cassettes provide great flexibility for the NHEJ process to generate successfully targeted events resulting in the production of functional marker proteins that can be visually screened for red fluorescence (Figure S5b,c).

**Figure 5:**
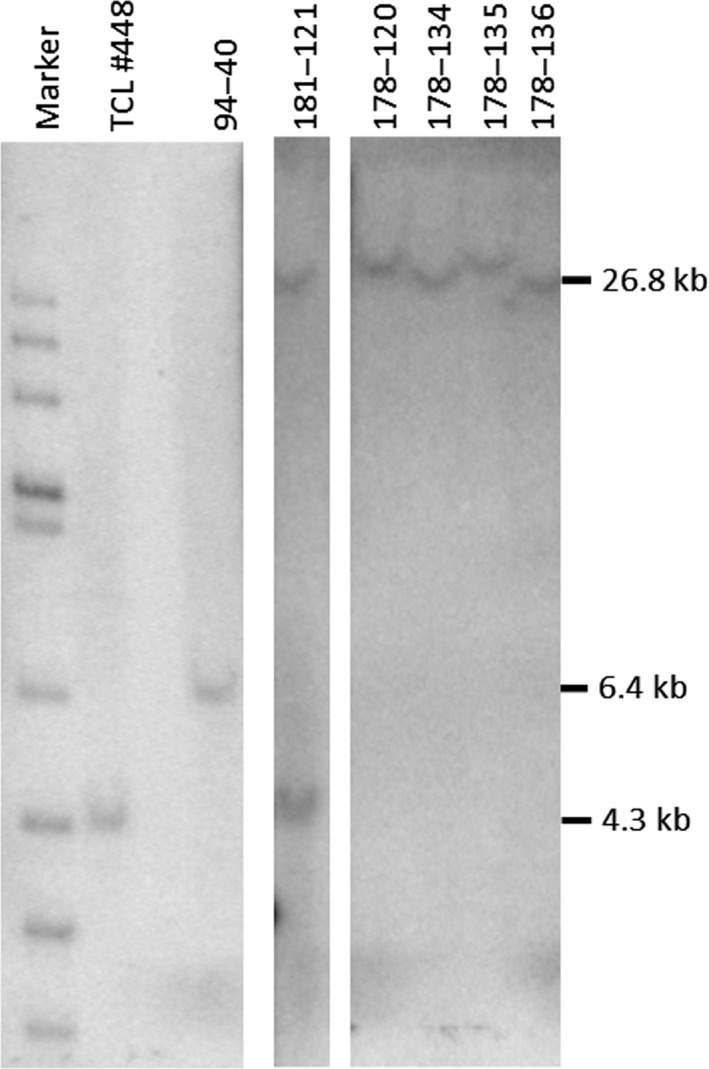
Southern blot analysis of events targeted with large donor vectors (payload 20 kb). Genomic DNA was digested with *Sac*I, and the blot was probed with the TR specific probe. As controls, the gDNA from the TCL#448 yielding a fragment of 4.3 kb and event 94‐40 targeted with a donor vector without additional payload, yielding a fragment of 6.4 kb were used

Subsequently, a Southern blot analysis was conducted on selected NHEJ‐mediated events with the largest donor construct (20‐kb payload). The entire ETIP was released by *Sac*I digestion, and resulting fragments were detected with the TR intron probe (Figure 1d). The results (Figure 5) confirm NGS data as only one fragment of a size of approx. 26 kb is detected. The size varied among the different NHEJ events reflecting deletions/integrations at the junction between target and donor DNA.

Table 2 summarizes the results for the integration of each of the eight different donor vectors at the target site. Many events contained both single targeted insertions and, additional, random donor DNA integrations (some of them partial) elsewhere in the genome. Most of these data were generated in experiments using only one ZFN, that is, ZFN2 cutting on the *npt*II side of the ETIP cassette. When both ZFNs were used, the targeting efficiency increased (44% to 82%) for NHEJ‐mediated targeting, while no effect is shown for HDR‐mediated targeting.

## 4 DISCUSSION

With the development of the ETIP strategy (Figure 1) and the establishment of corresponding tobacco BY‐2 target cell lines, we provide a versatile experimental system for targeted integration of genetic constructs into the tobacco genome. The convenient phenotypic selection/screening of putative targeted events greatly reduces the number of events that need to be analyzed in‐depth at the molecular level. The meticulous characterization of potential BY‐2 TCLs ensured the identification of events with only a single copy integration of the ETIP cassette per genome at a locus that allows reliable gene expression (Figure S2). Hence, these TCLs will enable the introduction of a single copy of a GOI at this defined locus. This concept will therefore allow the direct side by side comparison of different genetic constructs in terms of expression levels or efficacy of gene products without any interference originating from position effects (van Leeuwen, Mlynarova, Nap, van der Plas, & van der Krol, 2001), gene dosage (Beaujean, Sangwan, Hodges, & Sangwan‐Norreel, 1998), or silencing (Müller, 2010).

The deployment of intron sequences for splicing of the split markers (Puchta, Dujon, & Hohn, 1996) enables the introduction of donor DNA based either on HDR‐ or NHEJ‐mediated DSB repair mechanisms. DSB repair in somatic cells is mainly achieved by NHEJ‐mediated mechanisms (Knoll, Fauser, & Puchta, 2014) but HDR is activated simultaneously (Weimer et al., 2016). That both repair mechanisms can indeed act in parallel is apparent for event 88‐139 where the homology containing donor DNA has been integrated by HDR on the *npt*II side while the *DsRed* marker has been reconstituted by NHEJ (Figure 2b,c). Further, this example demonstrates that intron sequences can tolerate insertion of up to 3 kB without affecting their functionality. This conclusion is also supported by the analysis of events that have been generated through NHEJ (62‐65, 63‐16, 63‐49, 2‐25, 4‐12, and 5‐8) where the TR intron (*npt*II side) harbors vector backbone sequences (Figure 3a,c).

The selection/screening process provides a robust prediction of target donor DNA integration at both ends of the targeting site in the ETIP cassette. Therefore, the arbitrary selection of twenty events positive for both markers of a typical targeting experiment is sufficient to identify events with a single HDR‐mediated targeted integration without additional random integration of the donor DNA elsewhere in the genome. For the donor DNA including homology arms, ten out of 18 clones analyzed by Southern blot (Figure 2b‐d) are regarded as such clean events. One of these events (88‐139) had vector backbone donor DNA as described above, while the remaining clones had incorporated additional donor DNA copies. Although all events displayed the correct 3.2‐kb *Spe*I fragment or 3.3‐kb *Bam*HI/*Spe*I fragment on the 5′ or 3′ end, respectively, five clones (88‐143, 88‐163, 94‐20, 94‐21, and 94‐66) did not display the 6.4‐kb *Sac*I/*Sac*I fragment corresponding to a complete insertion of the donor DNA at the target site (Figure 2d). A possible explanation for this is that HDR has been initiated with independent donor DNA molecules at each end resulting in a complex integration pattern at the target site.

Targeted integration via a NHEJ‐mediated mechanism required the addition of ZFN recognition sites in the donor DNA (Figure 1b) to achieve either complete release of the engineered cassette or linearization of the vector molecule to generate ligation‐competent molecules in vivo (Cristea et al., 2013). Using this strategy, successful gene targeting has been demonstrated (Figure 3) although the additional integration of vector backbone sequence occurred more frequently, for example, events 62‐4, 62‐65, 63‐16, 63‐49 (Figure 3a) or 2‐25, 4‐12, 5‐8 (Figure 3c), indicating only single ZFN cleavage of the donor vector. Similarly, when the target DNA is only cleaved once, one intact ZFN binding site and the 130 bp spacer sequence between the recognition sites of ZFN2 and ZFN4 (Figure 1a) remain in the final recombined molecule, for example, events 3‐8 and 18‐12 (Figure 3c). The background of randomly inserted donor DNA as revealed by Southern blot analysis using the *DsRed*_5′ probe (Figure 3b,d) is higher compared with the HDR approach (Figure 2b). This is most likely a consequence of the linearization of the donor vector for NHEJ‐mediated targeting at the ZFN, which is prerequisite for targeting in this system while the donor vector for HDR‐mediated targeting does not require ZFN cleavage. The linear molecule can integrate wherever a naturally occurring DSB is encountered in the genome (Salomon & Puchta, 1998). A strategy to reduce the recovery of events with randomly integrated donor DNA copies would be the introduction of a negative selection marker (Thykjaer et al., 1997) distal to the ZFN recognition sites. Alternatively, when the ETIP platform is deployed in plant systems with sexual reproduction, randomly inserted donor DNA copies could be segregated from the targeted donor DNA copy in subsequent generations.

To evaluate targeting of large DNAs in plants using the ETIP platform for the targeted integration of large DNA molecules, a panel of vectors harboring payloads ranging from 5 kb to 20 kb was prepared. For all these vectors, designed either for HDR‐ or NHEJ‐mediated targeting, events with full‐size, single copy donor DNA integrated into the target site were identified (Table 2, Figure 5). Sequence analysis by NGS further indicated a high fidelity of donor DNA integration as most integrated copies contain no SNPs. For most of these targeting experiments, only one zinc finger nuclease (ZFN2) has been used as previous experiments indicated that integration of vector backbone DNA can be tolerated. Only for the largest vectors with 20‐kb payloads, a direct comparison has been made using either only ZFN2 alone or both zinc finger nucleases together. For the vector designed for HDR‐mediated integration, no differences were observed between these two approaches. In contrast, for the vector designed for NHEJ‐mediated integration, a considerably increased success rate for single copy targeted integration was noticed when both ZFNs were provided. Using both ZFNs for NHEJ‐mediated targeting enables the removal of the vector backbone from the donor DNA and therefore an almost seamless integration of the missing parts of both marker genes without additional unnecessary DNA in the introns. When only a single ZFN is used, the 3‐kb vector backbone needs to be integrated at the target site that might compromise the functionality of the intron sequence in certain instances.

Evidently even with the largest vectors, the size limit for successful targeting has not been reached, as the identification of single targeted events was easily possible, leaving room for payload sizes beyond 20 kb. In fact, the maximum size of donor DNA successfully integrated into the ETIP was 25 kb as the NHEJ vector designed with 20‐kb payload has been fully integrated after linearization with ZFN2 (Figure 4).

With the current ETIP system, we have designed a flexible targeting platform that can be used to address both fundamental questions about the recombination procedure itself and the evaluation of specific genetic constructs. Due to its large capacity, the system should facilitate the targeted integration of multiple expression cassettes, for example, coding for metabolic pathways (Farré et al., 2014) or multi‐subunit protein complexes, for example, mucosal antibodies (Vasilev, Smales, Schillberg, Fischer, & Schiermeyer, 2016). The upper size limit for the payload needs to be evaluated and might not be determined by the cell's capacity to integrate large DNA molecules but by the stability of these DNA molecules during the preparation and delivery process (Lengsfeld & Anchordoquy, 2002).

## CONFLICT OF INTEREST

K.J., R.B., P.M., P.S., D.R.C, S.R.W., D.O.G., W.M.A., and O.F. were employed by Dow AgroSciences LLC, a wholly owned subsidiary of The Dow Chemical Company, at the time the work was performed, and Dow AgroSciences provided funding for the research. Dow AgroSciences is now part of Corteva Agrisciences, which has filed a patent application on the ETIP concept and the cell lines containing the targeting cassette. All authors declare no conflict of interest.

## AUTHORS CONTRIBUTIONS

AS, DRC, SRW, RF, WMA, and SS designed the research; AS, KS, JK, TS, NK, KJ, DH, PM, and HS performed the research; AS, KS, RB, PS, DOG, OF, HS, and WMA analyzed the data; AS, JK, KS, PM, HS, and WMA wrote the paper.
