# Supplementary material for: Targeted insertion of large DNA sequences by homology‐directed repair or non‐homologous end joining in engineered tobacco BY‐2 cells using designed zinc finger nucleases
Source: Plant Direct. 2019 Jul 19;3(7):e00153. doi: 10.1002/pld3.153 (PMC6639735; doi:10.1002/pld3.153)

**Figure S1**: Functionality assay for partial and intron-containing marker genes. (a) Recombinant agrobacteria containing binary vectors carrying either the 5´ part, or 3´ part, or the intron-containing marker gene *DsRed* were infiltrated into *Nicotiana* *benthamiana* leaves to assess the phenotype. (b) Recombinant agrobacteria containing binary vectors carrying either the 5´ part, or 3´ part, or the intron-containing marker gene *nptII* were used to transform BY-2 cells and the cells were plated on kanamycin containing MS agar plates. Growth of transformed BY-2 cells was assessed after four weeks of cultivation.


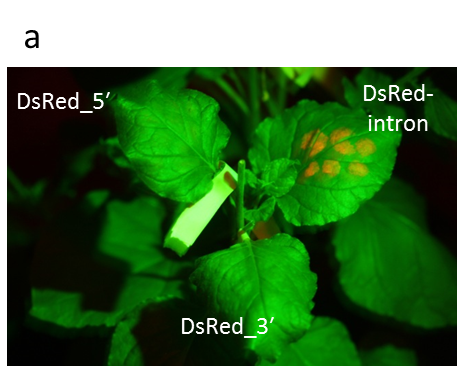


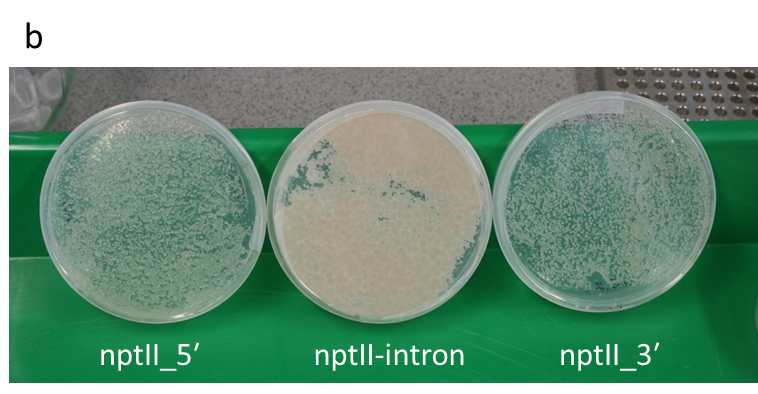

Supplement: Supplementary file 1 [file PLD3-3-e00153-s001.docx]
