# Supplementary material for: Targeted insertion of large DNA sequences by homology‐directed repair or non‐homologous end joining in engineered tobacco BY‐2 cells using designed zinc finger nucleases
Source: Plant Direct. 2019 Jul 19;3(7):e00153. doi: 10.1002/pld3.153 (PMC6639735; doi:10.1002/pld3.153)
Supplement: Supplementary file 2 [file PLD3-3-e00153-s002.docx]

**Figure S2**: Establishment of BY-2 target cell lines. (a) A schematic overview of the selection process. (b) An example of the Southern blot analysis probing the 3´ portion of the target construct. (c) The ranking of the target cell lines for their GFP fluorescence intensity relative to BY-2 cell line C#86. (d) NGS results (circus plot) showing integration of ETIP into the tobacco genome in TCL#448. Red, ETIP pDAB113628; green, tobacco genome scaffold Ntab-BX_AWOK-SS2405; blue, NGS coverage of ETIP from sequence capture.
