# Supplementary material for: Targeted insertion of large DNA sequences by homology‐directed repair or non‐homologous end joining in engineered tobacco BY‐2 cells using designed zinc finger nucleases
Source: Plant Direct. 2019 Jul 19;3(7):e00153. doi: 10.1002/pld3.153 (PMC6639735; doi:10.1002/pld3.153)

**Figure S3**: Southern blot of selected TCLs probing the 5´ part of the target construct. Genomic DNA was digested with *Xba*I and the DNA fragments hybridized with a probe recognizing the 5´ portion of the nptII marker gene.


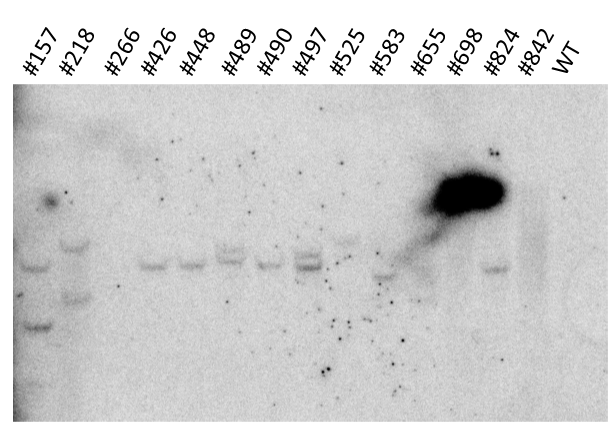

Supplement: Supplementary file 3 [file PLD3-3-e00153-s003.docx]
