# Supplementary material for: Targeted insertion of large DNA sequences by homology‐directed repair or non‐homologous end joining in engineered tobacco BY‐2 cells using designed zinc finger nucleases
Source: Plant Direct. 2019 Jul 19;3(7):e00153. doi: 10.1002/pld3.153 (PMC6639735; doi:10.1002/pld3.153)
Supplement: Supplementary file 4 [file PLD3-3-e00153-s004.docx]

**Figure S4**: Flow cytometry analysis of TCL#448 to evaluate stability of GFP-expression. Three separate BY-2 suspension cultures of TCL#448 were kept under a weekly subculture regime. Protoplasts from the three cultures were prepared and evaluated for GFP-fluorescence as described in experimental procedures. The percentage of GFP-expressing protoplasts among the analysed population was 98.3% for week 1 and 97.7% for week 13.
