# Supplementary material for: Targeted insertion of large DNA sequences by homology‐directed repair or non‐homologous end joining in engineered tobacco BY‐2 cells using designed zinc finger nucleases
Source: Plant Direct. 2019 Jul 19;3(7):e00153. doi: 10.1002/pld3.153 (PMC6639735; doi:10.1002/pld3.153)
Supplement: Supplementary file 5 [file PLD3-3-e00153-s005.docx]

**Figure S5**: DsRed fluorescence of HDR targeted events. BY-2 callus tissue was cultured on kanamycin selective MS agar medium at 26°C for 2 weeks prior DsRed fluorescence analysis using the ChemStudio PLUS imaging system (Analytic Jena, Germany) at a 480 nm excitation and an 607-682 nm emission filter with a 1 min exposure time. (a) HDR targeted callus tissue corresponding to Southern blot in Figure 2b. (b) NHEJ targeted callus tissue corresponding to Southern blot in Figure 5.
