# Supplementary material for: Targeted insertion of large DNA sequences by homology‐directed repair or non‐homologous end joining in engineered tobacco BY‐2 cells using designed zinc finger nucleases
Source: Plant Direct. 2019 Jul 19;3(7):e00153. doi: 10.1002/pld3.153 (PMC6639735; doi:10.1002/pld3.153)
Supplement: Supplementary file 6 [file PLD3-3-e00153-s006.docx]

**Figure S6**: Sequences of vectors used for HDR/NHEJ event generation. A) Vector used for the generation of TCLs (“target”); B) Donor vector used for HDR C) Donor vector used for NHEJ.

# A) >pDAB113628

ttgtacaaagtggttgcggccgcttgataagtaatttaaatttgctacataaggactaggtacagccccg

gcattgaacgctcgtacagtcctgatcgaatctggcttttccagaaacacgaacctagtagttttggtgc

agaccttagtcatacacaaccttcactggtatatcattgtgtttgattggactagcgagagtgactcttt

taattgtttctcgccgcaatgaaggagccatcacacgctgcgttgagcgccccagccgacgctaagcatg

ataacgactagcgatttgcgtgtggcttaagtcttggcatacctacgatcgttagtccacgttgtctgta

gaccatcggtattatgattctgcagtgtttacgatggtgatagccatgaaatgaaaaagggttcctgaca

tccaactgataagcctaaaagttgcaccagaagtaccagttaaggaggcctctataatggctagaataaa

gcagcgttgaacctcagagtcccaccaccgtgaccggcccttacgttcgccggaattgtccagagtctgg

taaatcgtgtaggggaggaaacgagcgcctcccggaacggatttagtgcgtccctcagcagccgtgtacg

cctctgtgtgaccccgtaacagtctatttaccagttttaggtactctcgttgctaggcccgccagtgtgt

aaaagttggtgaaatcactaggcggtctcaatgcgtcgcagaccctacgtggaaggcggaatttcagcat

gagtgtgacgttcgatgatcgctcaaatcgtttaaacagcgatcggcggtaatgaaaccctccacataat

aggaccactagggccgcgcatttatgagagtagcaaggcaagcattggatgaggttcaagccttctttcc

tgtaagacgttctttatacagttagctccgagtggggagtcacgggtggtgtcacggctaacaggggtcg

gtgtacctcgacttattaagtattcccgctcaaactgactggggtgacaagttaggaaagtatgcataga

tgcactcgaaatcagccaattttagacaagtatcaaacggatgtgacttcagtacattaaaaacgtccgc

aatgtgttattaagttgtctaagcgtcaatttgatttacaattgaatatatcctgccccagccagccaac

agctcgatttacaattgaatatatcctgccggccggcccacgcgtgtcgaggaattctgatctggccccc

atttggacgtgaatgtagacacgtcgaaataaagatttccgaattagaataatttgtttattgctttcgc

ctataaatacgacggatcgtaatttgtcgttttatcaaaatgtactttcattttataataacgctgcgga

catctacatttttgaattgaaaaaaaattggtaattactctttctttttctccatattgaccatcatact

cattgctgatccatgtagatttcccggacatgaagccatttacaattgaatatatcctgccgccgctgcc

gctttgcacccggtggagcttgcatgttggtttctacgcagaactgagccggttaggcagataatttcca

ttgagaactgagccatgtgcaccttccccccaacacggtgagcgacggggcaacggagtgatccacatgg

gacttttaaacatcatccgtcggatggcgttgcgagagaagcagtcgatccgtgagatcagccgacgcac

cgggcaggcgcgcaacacgatcgcaaagtatttgaacgcaggtacaatcgagccgacgttcacgcggaac

gaccaagcaagcttggctgccatttttggggtgaggccgttcgcggccgaggggcgcagcccctgggggg

atgggaggcccgcgttagcgggccgggagggttcgagaagggggggcccccttcggcgtgcgcggtcacg

cgcacagggcgcagccctggttaaaaacaaggtttataaatattggtttaaaagcaggttaaaagacagg

ttagcggtggccgaaaaacgggcggaaacccttgcaaatgctggattttctgcctgtggacagcccctca

aatgtcaataggtgcgcccctcatctgtcagcactctgcccctcaagtgtcaaggatcgcgcccctcatc

tgtcagtagtcgcgcccctcaagtgtcaataccgcagggcacttatccccaggcttgtccacatcatctg

tgggaaactcgcgtaaaatcaggcgttttcgccgatttgcgaggctggccagctccacgtcgccggccga

aatcgagcctgcccctcatctgtcaacgccgcgccgggtgagtcggcccctcaagtgtcaacgtccgccc

ctcatctgtcagtgagggccaagttttccgcgaggtatccacaacgccggccggccgcggtgtctcgcac

acggcttcgacggcgtttctggcgcgtttgcagggccatagacggccgccagcccagcggcgagggcaac

cagcccggtgagcgtcggaaagggtcgacggatcttttccgctgcataaccctgcttcggggtcattata

gcgattttttcggtatatccatcctttttcgcacgatatacaggattttgccaaagggttcgtgtagact

ttccttggtgtatccaacggcgtcagccgggcaggataggtgaagtaggcccacccgcgagcgggtgttc

cttcttcactgtcccttattcgcacctggcggtgctcaacgggaatcctgctctgcgaggctggccggct

accgccggcgtaacagatgagggcaagcggatggctgatgaaaccaagccaaccaggaagggcagcccac

ctatcaaggtgtactgccttccagacgaacgaagagcgattgaggaaaaggcggcggcggccggcatgag

cctgtcggcctacctgctggccgtcggccagggctacaaaatcacgggcgtcgtggactatgagcacgtc

cgcgagctggcccgcatcaatggcgacctgggccgcctgggcggcctgctgaaactctggctcaccgacg

acccgcgcacggcgcggttcggtgatgccacgatcctcgccctgctggcgaagatcgaagagaagcagga

cgagcttggcaaggtcatgatgggcgtggtccgcccgagggcagagccatgacttttttagccgctaaaa

cggccggggggtgcgcgtgattgccaagcacgtccccatgcgctccatcaagaagagcgacttcgcggag

ctggtattcgtgcagggcaagattcggaataccaagtacgagaaggacggccagacggtctacgggaccg

acttcattgccgataaggtggattatctggacaccaaggcaccaggcgggtcaaatcaggaataagggca

cattgccccggcgtgagtcggggcaatcccgcaaggagggtgaatgaatcggacgtttgaccggaaggca

tacaggcaagaactgatcgacgcggggttttccgccgaggatgccgaaaccatcgcaagccgcaccgtca

tgcgtgcgccccgcgaaaccttccagtccgtcggctcgatggtccagcaagctacggccaagatcgagcg

cgacagcgtgcaactggctccccctgccctgcccgcgccatcggccgccgtggagcgttcgcgtcgtctc

gaacaggaggcggcaggtttggcgaagtcgatgaccatcgacacgcgaggaactatgacgaccaagaagc

gaaaaaccgccggcgaggacctggcaaaacaggtcagcgaggccaagcaggccgcgttgctgaaacacac

gaagcagcagatcaaggaaatgcagctttccttgttcgatattgcgccgtggccggacacgatgcgagcg

atgccaaacgacacggcccgctctgccctgttcaccacgcgcaacaagaaaatcccgcgcgaggcgctgc

aaaacaaggtcattttccacgtcaacaaggacgtgaagatcacctacaccggcgtcgagctgcgggccga

cgatgacgaactggtgtggcagcaggtgttggagtacgcgaagcgcacccctatcggcgagccgatcacc

ttcacgttctacgagctttgccaggacctgggctggtcgatcaatggccggtattacacgaaggccgagg

aatgcctgtcgcgcctacaggcgacggcgatgggcttcacgtccgaccgcgttgggcacctggaatcggt

gtcgctgctgcaccgcttccgcgtcctggaccgtggcaagaaaacgtcccgttgccaggtcctgatcgac

gaggaaatcgtcgtgctgtttgctggcgaccactacacgaaattcatatgggagaagtaccgcaagctgt

cgccgacggcccgacggatgttcgactatttcagctcgcaccgggagccgtacccgctcaagctggaaac

cttccgcctcatgtgcggatcggattccacccgcgtgaagaagtggcgcgagcaggtcggcgaagcctgc

gaagagttgcgaggcagcggcctggtggaacacgcctgggtcaatgatgacctggtgcattgcaaacgct

agggccttgtggggtcagttccggctgggggttcagcagccagcgctttactggcatttcaggaacaagc

gggcactgctcgacgcacttgcttcgctcagtatcgctcgggacgcacggcgcgctctacgaactgccga

taaacagaggattaaaattgacaattgtgattaaggctcagattcgacggcttggagcggccgacgtgca

ggatttccgcgagatccgattgtcggccctgaagaaagctccagagatgttcgggtccgtttacgagcac

gaggagaaaaagcccatggaggcgttcgctgaacggttgcgagatgccgtggcattcggcgcctacatcg

acggcgagatcattgggctgtcggtcttcaaacaggaggacggccccaaggacgctcacaaggcgcatct

gtccggcgttttcgtggagcccgaacagcgaggccgaggggtcgccggtatgctgctgcgggcgttgccg

gcgggtttattgctcgtgatgatcgtccgacagattccaacgggaatctggtggatgcgcatcttcatcc

tcggcgcacttaatatttcgctattctggagcttgttgtttatttcggtctaccgcctgccgggcggggt

cgcggcgacggtaggcgctgtgcagccgctgatggtcgtgttcatctctgccgctctgctaggtagcccg

atacgattgatggcggtcctgggggctatttgcggaactgcgggcgtggcgctgttggtgttgacaccaa

acgcagcgctagatcctgtcggcgtcgcagcgggcctggcgggggcggtttccatggcgttcggaaccgt

gctgacccgcaagtggcaacctcccgtgcctctgctcacctttaccgcctggcaactggcggccggagga

cttctgctcgttccagtagctttagtgtttgatccgccaatcccgatgcctacaggaaccaatgttctcg

gcctggcgtggctcggcctgatcggagcgggtttaacctacttcctttggttccgggggatctcgcgact

cgaacctacagttgtttccttactgggctttctcagcccccgagcgcttagtgggaatttgtacccctta

tcgaaccgggagcacaggatgacgcctaacaattcattcaagccgacaccgcttcgcggcgcggcttaat

tcaggagttaaacatcatgagggaagcggtgatcgccgaagtatcgactcaactatcagaggtagttggc

gtcatcgagcgccatctcgaaccgacgttgctggccgtacatttgtacggctccgcagtggatggcggcc

tgaagccacacagtgatattgatttgctggttacggtgaccgtaaggcttgatgaaacaacgcggcgagc

tttgatcaacgaccttttggaaacttcggcttcccctggagagagcgagattctccgcgctgtagaagtc

accattgttgtgcacgacgacatcattccgtggcgttatccagctaagcgcgaactgcaatttggagaat

ggcagcgcaatgacattcttgcaggtatcttcgagccagccacgatcgacattgatctggctatcttgct

gacaaaagcaagagaacatagcgttgccttggtaggtccagcggcggaggaactctttgatccggttcct

gaacaggatctatttgaggcgctaaatgaaaccttaacgctatggaactcgccgcccgactgggctggcg

atgagcgaaatgtagtgcttacgttgtcccgcatttggtacagcgcagtaaccggcaaaatcgcgccgaa

ggatgtcgctgccgactgggcaatggagcgcctgccggcccagtatcagcccgtcatacttgaagctagg

caggcttatcttggacaagaagatcgcttggcctcgcgcgcagatcagttggaagaatttgttcactacg

tgaaaggcgagatcaccaaggtagtcggcaaataatgtctaacaattcgttcaagccgacgccgcttcgc

ggcgcggcttaactcaagcgttagagagctggggaagactatgcgcgatctgttgaaggtggttctaagc

ctcgtcttgcgatggcatttcgatccattcccattccgcgctcaagatggcttcccctcggcagttcatc

agggctaaatcaatctagccgacttgtccggtgaaatgggctgcactccaacagaaacaatcaaacaaac

atacacagcgacttattcacacgagatcaaattacaacggtatatatcctgccagtcagcatcatcacac

caaaagttaggcccgaatagtttgaaattagaaagctcgcaattgaggtctacaggccaaattcgctctt

agccgtacaatattactcaccagatcctaaccggttgatatggcacgcaacatttcgcagctgatgcaga

ggtcatctcgctgcacaagatatgcaaccataaaacgctagattacacttttgcttaaatgcccgtttaa

accgtggagagtaaaatgcagtcaactctctataccaaacattgttgacattcttgggtacgtttgcgct

cagaacgcccaccttgcctccttaggtaagattatccaacggctgtctcccatattatcgtctccacgtg

tgattgtgccaatcatagcgggaacgccttagcgcgaccctgtcgcctgttaagaatttcggcaaacttg

cttccatccctcgtttcagtgatgctcattagacctacacccaaaaaccagattatggttgttagccaag

ctgcgtctcgccgtttgtagctatttttgcacgccccatgagcccatgagtacggcaatcagtcgcctgg

gtgactaccaagtccggtatacggggtttaatcgtgctggtacgccttgggaggcgtaacatggcatgac

ttttcgttgacaagagctggcaacgaaagaggggtgtgcctgggattagactccacaacaggcggagggt

cgaaaaatttgtgtgtaacgaaatttgaaggagtgctatataacccactagtaccctctccgtattgtcg

atgcgggcctgccctcgacaaggtaatgcggtcacctggtgagctcaaacgtcatgggataaacccctcc

cttagtttatagccgaatatgtcacgacgctcactggtaatatgtctgatgctttgctcgccgtcagtcc

gtctgccaggtttctggtatgagattttccaacgtgcttagttccaaaaggcgtcgcaggaggccaattt

cagaaagatggattgtccctgctttatcaataatgaatacaaggaactatgggtcatccgacggggtaaa

cagtgagtggctcgttagtcgaggtatcaacagcgtataattttcgtgccattgtgcggccgcacaagtt

tgtacaaaaaagcaggctttaaaggaaccaattcagtcgacgcaggctttaaaggaaccaattcagggcg

cgccaagcttagatctttaattaataggtcggattgcgtctgctcatcgatcatgagcggagaattaagg

gagtcacgttatgacccccgccgatgacgcgggacaagccgttttacgtttggaactgacagaaccgcaa

cgttgaaggagccactcagccgcgggtttctggagtttaatgagctaagcacatacgtcagaaaccatta

ttgcgcgttcaaaagtcgcctaaggtcactatcagctagcaaatatttcttgtcaaaaatgctccactga

cgttccataaattcccctcggtatccaattagagtctcatattcactctcaactcgatcgaggcatgatt

gaacaagatggattgcacgcaggttctccggccgcttgggtggagaggctattcggctatgactgggcac

aacagacaatcggctgctctgatgccgccgtgttccggctgtcagcgcaggggcgcccggttctttttgt

caagaccgacctgtccggtgccctgaatgaactccaagacgaggcagcgcggctatcgtggctggccacg

acgggcgttccttgcgcagctgtgctcgacgttgtcactgaagcgggaagggactggctgctattgggcg

aagtgccggggcaggatctcctgtcatctcaccttgctcctgccgagaaagtatccatcatggctgatgc

aatgcggcggctgcatacgcttgatccggctacctgcccattcgaccaccaagcgaaacatcgcatcgag

cgaggacgtactcggatggaagccggtcttgtcgatcaggatgatctggacgaagagcatcaggggctcg

cgccagccgaactgttcgccaggctcaaggcgcggatgcccgacggcgaggatctcgtcgtgacccaggg

cgatgcctgcttgccgaatatcatggtggaaaatggccgcttttcaggtaagtttgtggattcttcgtcc

atgtgatctttgagtttctttagagcttgtgagggattagtaagtaacaatgcttgagttttttgctgct

gggcttcgaaaagtttgtcacttgttggtttgatccacaaggtcttcttctccatagctactagacatgt

tttagcttaagattcaagtttatatatgccttgtggattaatcattgcctgattcttccgtgtcatctct

gagtttatttagagcttggaagtggtgtagtaataactaacaatactcttgataagttgtagcaatgctc

ttgattagtggatgtaatatgatgttgataagatatatgaggcacagaaccaaaagtggtgcttccacta

gacccgtttttagcctaaggttcaagtttataccttgtagatgtttctgtattgtctgattcttccctgt

gatatttgaatttcttagagctttggaagtgatataggaacaatgctcttgtgtgtttgtctctatgaag

attatcgctgtcgtgtttcatccgagtgtgcgggattttttgctgctgggtttagcctttcttcaaaaag

ttattacttgttagttttattgttttggtcttgataagagatgttaggacagacatggtgcttcttgtct

atagccactagacctattttagcataaggttaacgaaattatgcaaagactcccgcccatagatctatgc

ccgggacaagtggagccacaggttagtagagtggtaggagcagtgttggacgcagagtaccgcctatggg

tttgatggctcgcttacataagcagttctgtcccatggagccaatgtcctgatacggtaggacacatatc

ccgccttttgcagtttaagctttatgcccgggacaagtgaattcagtagttttgatcatacacacgtttc

atgtggtacttgagagttactaatttttgtcatcttcgtataagtagtaaaagatactacaagaatagtt

tagtagaaaatactagcggtaggtgaagatttgtcgctatgtactattattgtctagtaacttgagtaac

aatttcgtggtctaaatatcaaataaaaatggatgagtggttcaccaaatctaggcatcaaaactattaa

tgtcattgtctagatcttaggtgacaccacatttcgaatatttattggtaattgagatgttaaagtacca

atatttgacttaataaactaaaagattttggctttatcaaatgtagacattgatgacatatcgttgtcat

tatcttgagtatatacaagtcgatcaattaggtgaaagtttagtgtctcgtggttggtaaacgattaata

cagtagtatattttatccaaagacaaaatccaaatcatttcaccagtatgaatagtattattttatctta

aaagctaaaatcttaaaaaccaaggtagcacccacgttgagctagacgatcaaatcgatttctgctttgt

ccaatttaccaagctatttaaagccaaataattgaaatataggtaggtcgttatattaggctaagattta

tctcaaatgcttaactaaaggaataacaagggattctagttgtgtggttttataagattggtccaatttc

acttaagtttgtttattgtagaattttatatgtgaataatttgaattccaattgaaaagatattatagta

aaagaaaaaatagtgcgaacaaaaaactttaatcccataaaaagaaaaagaaaaatgaaaagttcttcta

acatccatattttgcatcatatcataaagataagaaagatacatatcatagacgtacagataaacaaaca

tatcatcatttgtgaaatacatagtacaataatttgcttttaaatagagtttaagtcacacacactgaca

cacacgataaaacgataatgtctgcaaaaacactttaatcccattgcctagaggacagcttctccacttt

gtctttaaggttggttttgccgtgttgtttttatctttatataatgatctattttttggattatgaaatg

aattcacacattttaattatttaagaagatccatatacaggtttataacagtactaagtgatgattattt

tttgtttttgcatagtttagtttattgggtaaacattcattacgtgtctctttatacgaatcacccatcc

aaaatttcaagtagtcttttagttcatttattatttcataactatttgacttattgatttgacaagaaac

aacaaaagtgttgacttattgatagattgtgggatcataaaagtaattaagcgtcaaccacgacccacaa

caacaaagcacatgttatacattaatatctcgtttacttaattacagttttcagaatgccgtttcatgtc

ttgtcactggcgatgttattatcatgttggacaatattcgactgttgtcgtttttacattttcgtattga

ctaaaactaaaaaaacaaaactctgtttcaggtatatgtcaagcaccctgccgacataccagactataaa

aagctgtcatttcctgaaggatttaaatgggaaagggtcatgaactttgaagacggtggcgtcgttactg

taacccaggattccagtttgcaggatggctgtttcatctacaaggtcaagttcattggcgtgaactttcc

ttccgatggacctgttatgcaaaagaagacaatgggctgggaagccagcactgagcgtttgtatcctcgt

gatggcgtgttgaaaggagagattcataaggctctgaagctgaaagacggtggtcattacctagttgagt

tcaaaagtatttacatggcaaagaagcctgtgcagctaccagggtactactatgttgactccaaactgga

tataacaagccacaacgaagactatacaatcgttgagcagtatgaaagaaccgagggacgccaccatctg

ttccttgcggccgctcatcaccatcaccatcactctgagaaagatgagctctagctcgagctcggatcct

ctagagtccgcaaaaatcaccagtctctctctacaaatctatctctctctatttttctccagaataatgt

gtgagtagttcccagataagggaattagggttcttatagggtttcgctcatgtgttgagcatataagaaa

cccttagtatgtatttgtatttgtaaaatacttctatcaataaaatttctaattcctaaaaccaaaatcc

agtgaccgtgcacaagcttcggatttggagccaagtctcataaacgccattgtggaagaaagtcttgagt

tggtggtaatgtaacagagtagtaagaacagagaagagagagagtgtgagatacatgaattgtcgggcaa

caaaaatcctgaacatcttattttagcaaagagaaagagttccgagtctgtagcagaagagtgaggagaa

atttaagctcttggacttgtgaattgttccgcctcttgaatacttcttcaatcctcatatattcttcttc

tatgttacctgaaaaccggcatttaatctcgcgggtttattccggttcaacattttttttgttttgagtt

attatctgggcttaataacgcaggcctgaaataaattcaaggcccaactgttttttttttaagaagttgc

tgttaaaaaaaaaaaaagggaattaacaacaacaacaaaaaaagataaagaaaataataacaattacttt

aattgtagactaaaaaaacatagattttatcatgaaaaaaagagaaaagaaataaaaacttggatcaaaa

aaaaaaacatacagatcttctaattattaacttttcttaaaaattaggtcctttttcccaacaattaggt

ttagagttttggaattaaaccaaaaagattgttctaaaaaatactcaaatttggtagataagtttcctta

ttttaattagtcaatggtagatacttttttttcttttctttattagagtagattagaatcttttatgcca

agtattgataaattaaatcaagaagataaactatcataatcaacatgaaattaaaagaaaaatctcatat

atagtattagtattctctatatatattatgattgcttattcttaatgggttgggttaaccaagacatagt

cttaatggaaagaatcttttttgaactttttccttattgattaaattcttctatagaaaagaaagaaatt

atttgaggaaaagtatatacaaaaagaaaaatagaaaaatgtcagtgaagcagatgtaatggatgaccta

atccaaccaccaccataggatgtttctacttgagtcggtcttttaaaaacgcacggtggaaaatatgaca

cgtatcatatgattccttcctttagtttcgtgataataatcctcaactgatatcttcctttttttgtttt

ggctaaagatattttattctcattaatagaaaagacggttttgggcttttggtttgcgatataaagaaga

ccttcgtgtggaagataataattcatcctttcgtctttttctgactcttcaatctctcccaaagcctaaa

gcgatctctgcaaatctctcgcgactctctctttcaaggtatattttctgattctttttgtttttgattc

gtatctgatctccaatttttgttatgtggattattgaatcttttgtataaattgcttttgacaatattgt

tcgtttcgtcaatccagcttctaaattttgtcctgattactaagatatcgattcgtagtgtttacatctg

tgtaatttcttgcttgattgtgaaattaggattttcaaggacgatctattcaatttttgtgttttctttg

ttcgattctctctgttttaggtttcttatgtttagatccgtttctctttggtgttgttttgatttctctt

acggcttttgatttggtatatgttcgctgattggtttctacttgttctattgttttatttcagccatggc

ggctgccacttcgaactccgctttgcctaagctttctacgttaacttcgtccttcaaatcttccataccc

atttccaaatccagccttcccttctccacaacccctcaaaagcccaccccttaccgctccttcgacgttt

cctgctctctttctcatgcaagctccaaccccagatccgctgccgcatccgttacccaaaaaactgctcc

tccccattatttcatttccaggtatgcggatgatgagccccggaaaggcgctgatatcctggtggaagcc

ctggaacgtgaaggggtcaaggatgtgtttgcctacccaggtggagcttcaatggagatccaccaggctt

taacccgctcaaaaatcatccgaaatgtccttccgcgacacgagcaaggtggggtctttgccgccgaggg

ttacgcgcgctcctctggcatttccggcgtttgcattgcgacgtctggccctggggcaaccaacttggtg

agtggtctcgctgatgcgatgctcgatagtatccctctcgtggcgatcactggtcaagtccctcgtcgga

tgatcggtaccgatgctttccaggaaactccaattgttgaggtaacaaggtctattacgaagcataatta

tcttgttcttgatgtggatgatattcctaggattgttagtgaggctttctttttagcttcatcgggcagg

ccgggacctgttctgattgatgttcctaaggatatacaacagcaacttgctgttcctaaatggaaccatt

ctcttagattgccagggtatttgtctaggttgcctaaggctcccgctgaggctcatctcgaacagatcgt

gagattggtttctgagtctaagaagcctgttttatatgttggtggtgggtgtttgaactctagtgaggag

ttgaagaggtttgttgagcttacagggatacccgttgcaagtactttgatgggtcttggagcctttccga

tttcggatgagttgtcgttacaaatgcttgggatgcacggaactgtgtatgccaattatgctgttgataa

gagtgatttgttgcttgcttttggagtgagatttgatgatagggtgacaggaaaacttgaggcttttgcc

agccgggcaaagattgtgcatatcgatatcgactctgctgagattgggaagaacaagcagcctcatatgt

cagtgtgttccgatgtgaaattggcattgcaggggataaataagatattggagaccacgggagctaagct

gaatcttgattattcggaatggaggcaggagttaaacgagcagaagctgaaattccctttgagttacaag

acctttggtgaagctattccacctcaatatgcaattcaggttcttgatgaattaactggcgggaatgcaa

ttataagtaccggtgttggccagcatcaaatgtgggctgctcaattttacaagtataagaagcctcgtca

atggttaacgtctgggggattgggtgctatgggatttggattgcctgctgctattggagctgctgttgca

aacccggaggcagttgttgtagacatcgacggtgatggaagttttatcatgaatgtgcaagagttggcga

ctatccgtgtggaaaatcttccggttaagatattattgttgaataatcagcatttgggtatggttgttca

atgggaggaccggttttacaaggcaaacagggctcatacatacttgggagacccatccaacgagtcggaa

atattcccaaatatgttgaaatttgctgaagcatgcgggataccagctgcccgggtgacgaagaaagaag

atctcaaagcagcaattcagaaaatgttggacactcctggaccttacttgttggatgtgattgtcccaca

tcaagaacatgtcctgcctatgatccccaatggaggcgctttcaaagatgtgatcacagagggtgatgga

agaacacaatattgagagctctgtccaacagtctcagggttaatgtctatgtatcttaaataatgttgtc

ggtattttgtaatctcatatagattttcactgtgcgacgcaaaaatattaaataaatattattattatct

acgttttgattgagatatcatcaatattataataaaaatatccattaaacacgatttgatacaaatgaca

gtcaataatctgatttgaatatttattaattgtaacgaattacataaagatcgaatagaaaatactgcac

tgcaaatgaaaattaacacatactaataaatgcgtcaaatatctttgccaagatcaagcggagtgagggc

ctcatatccggtctcagttacaagcacggtatccccgaagcgcgctccaccaatgccctcgacatagatg

ccgggctcgacgctgaggacattgcctaccttgagcatggtctcagcgccggctttaagctcaatcccat

cccaatctgaatatcctatcccgcgcccagtccggtgtaagaacgggtctgtccatccacctctgttaca

gccactgcgccgcatggacctcacgtgccagggcggccgcacttagtggatttgttccacacgggagaaa

aggggacccctttcttccaccgttgatacacgttatgcacagagtccgcacttgggagaggccacccctt

gatgaaccagatggagggataccgttatcacgtatggtctgcctttgtacggaaagcagattgtggcagt

agctcagtctactcatactgcgagcggtcgtttggcctccgagacgaggtttcctacctggacgagccac

aatcgtagcacctagtcaaccttagacccgtgccgtctacttgcaaggggacattcgtagtgggatgcca

gacagagaccacattgatgctcctgcatctgcttccgtgtcctatacaagcctgaaggaatgatactgtt

gcctcacactaacatgtgtggtcatcctcatcctgataaactgcaaaaggccatatagtccccgtggcga

cacggcgcgccggtaggtaccgagctcttacgcgtgctagcccgggctggtagaatggttggaatgaaaa

aaattatattttctcaccgttcatattttataaggtggtgaagaaattattccaattgaatatttttttt

gtaattgtgtggacataatataaatttatgaatatttatgaattgaagaaaggcaaaggccacaagaggt

gaatgaaagcgatatcataaaaccaaaaaacacaaattcaattttcaaatttcaaaaaattgggggctcc

aattccaaattctcagcaagccgaagccgagcagaagccgaaaataaagatccaacggtggagattaaag

aaatgaaaaaagaggaaaaagaaaggaagaagaaaggaagaatggggctgggaaaggctgtcagccaggt

caccctatcttctctggtggtcgaaatgattccttctccaaatttctcatttccttcgcatttgcatttg

catttgcatttcccttctttccctctctctctctctctctctctctctctctctctgtttataaaccccc

gtttctcttcttccctcttcctcttattctcgtctttcaactcacctaggtcgacaacactcactcctct

ctcagccagaccttcttctttggagggttggctctttcttcttcgttcgttccttccttccttcattcat

tctcctctctttcatccaaggttgtttcttccttcccttttttaccaaatcttctcacttcccttacatt

tttcatctggggtatcgttcttttcccaaattatgctgctttcgtctctcatttatctactttattgctt

ttaactcattttcccttatgcggttcttcaattttggctgatcttgctgtttgttttggaattctgtttt

aatcgccctggatccgaggtttttgttcgtacaatctacctagattctttctgtttgtttgctgatctga

aattttccatttgggttttgattgtctgtgcttacggaactgagatctaggatttggagttgtgtacctt

tttatttctgcatgcaattctgtaatcctgcatagctggatggctttctgttgattagtgcatgctttgt

ttaggacgaactgacttggatttttcgttgtcgatctgttctattttttgttttgctgttctggttcatg

cttggaatgatttagttgctttgtaaattgtacactctgcttttgtgttagttcacgtagcttctcgatc

tgaaattggatatggttagagtttatggtcagcttgtgatcttgcattatgcaaaaattggaactttaat

ccttttcatttgtaagatctttaagatatctgattacctggttgatttttttgtgtctggattattttat

ttgttttgaaagtagtttgttggttcttcctgtattatttgctgaatcgggatgatcaattatatgacgt

gaatttatggaatgtaaatgaatggtttaagagattgctttgtgtggcttatttattcaatttctatttt

tacatcgttttgtgcaggttttgaaaaaaaagggcccatggctcgtgcacaacttgtccttgtggcattg

gttgcagctgctctgctcttggctggtcctcacaccacaatggctatggagtccgatgagagtggtctcc

cagctatggagattgaatgcagaatcactggcactttgaacggtgttgagtttgaactggtgggaggtgg

cgaagggacacctgaacaagggaggatgacaaacaagatgaagtccaccaaaggtgcattgaccttctct

ccgtatcttctcagccatgtcatgggttacggtttctatcactttggcacctatccgagtggctatgaga

atccctttcttcatgccatcaacaatggaggttacaccaacacacgaattgagaagtatgaagatggtgg

agtgctccacgtctccttctcttaccgttacgaggctgggagggtcataggagacttcaaagtgatggga

actggctttccagaagattcagtcatcttcacagacaagatcattagatccaatgcaactgttgagcatc

ttcacccaatgggagacaatgacctggatgggtcattcacaagaaccttctctctgcgtgatggaggcta

ctatagctctgttgtggactcacacatgcacttcaaaagtgccattcatcctagcatcttgcagaatggt

ggacccatgtttgcctttcgaagggtggaagaggatcactcaaacaccgaacttggcatagttgagtacc

agcatgccttcaagactcctgatgcagatgctggggaagagaaagatgaactgtgagtagttagcttaat

cacctagagctcgaatttccccgatcgttcaaacatttggcaataaagtttcttaagattgaatcctgtt

gccggtcttgcgatgattatcatataatttctgttgaattacgttaagcatgtaataattaacatgtaat

gcatgacgttatttatgagatgggtttttatgattagagtcccgcaattatacatttaatacgcgataga

aaacaaaatatagcgcgcaaactaggataaattatcgcgcgcggtgtcatctatgttactagatcgctgc

agtggtcatcctcatcctgataaactgcaaaaggctcacttagtggatttgttccacacgggagaaaagg

ggacccctttcttccaccgttgatacacgttatgcacagagtccgcacttgggagaggccaccccttgat

gaaccagatggagggataccgttatcacgtatggtctgcctttgtacggaaagcagattgtggcagtagc

tcagtctactcatactgcgagcggtcgtttggcctccgagacgaggtttcctacctggacgagccacaat

cgtagcacctagtcaaccttagacccgtgccgtctacttgcaaggggacattcgtagtgggatgccagac

agagaccacattgatgctcctgcatctgcttccgtgtcctatacaagcctgaaggaatgatactgttgcc

tcacactaacatgtgcagtgcgattcggcatcaatccaagcttttaattaaggcgcgccctcgagatatc

tagacccagctttc

# B) >pDAB113676

tcgagaagcttgatgcccgacggcgaggatctcgtcgtgacccagggcgatgcctgcttgccgaatatca

tggtggaaaatggccgcttttcagggtaagtttgtggattcttcgtccatgtgatctttgagtttcttta

gagcttgtgagggattagtaagtaacaatgcttgagttttttgctgctgggcttcgaaaagtttgtcact

tgttggtttgatccacaaggtcttcttctccatagctactagacatgttttagcttaagattcaagttta

tatatgccttgtggattaatcattgcctgattcttccgtgtcatctctgagtttatttagagcttggaag

tggtgtagtaataactaacaatactcttgataagttgtagcaatgctcttgattagtggatgtaatatga

tgttgataagatatatgaggcacagaaccaaaagtggtgcttccactagacccgtttttagcctaaggtt

caagtttataccttgtagatgtttctgtattgtctgattcttccctgtgatatttgaatttcttagagct

ttggaagtgatataggaacaatgctcttgtgtgtttgtctctatgaagattatcgctgtcgtgtttcatc

cgagtgtgcgggattttttgctgctgggtttagcctttcttcaaaaagttattacttgttagttttattg

ttttggtcttgataagagatgttaggacagacatggtgcttcttgtctatagccactagacctattttag

cataaggttaacgaaattatgcaaagactcccgcccatagactctctacataccttgtggatttgtttac

attgcctgatctttcctgtgatcgctgtcatgtttctttggaatgattgatgtttataaatggaaaaatc

tttgtgcaggtttcatcgactgtggccggctgggtgtggcggaccgctatcaggacatagcgttggctac

ccgtgatattgctgaagagcttggcggcgaatgggctgaccgcttcctcgtgctttacggtatcgccgct

cccgattcgcagcgcatcgccttctatcgccttcttgacgagttcttctgagcgggactctggggttcgg

actctagctagagtcaagcagatcgttcaaacatttggcaataaagtttcttaagattgaatcctgttgc

cggtcttgcgatgattatcatataatttctgttgaattacgttaagcatgtaataattaacatgtaatgc

atgacgttatttatgagatgggtttttatgattagagtcccgcaattatacatttaatacgcgatagaaa

acaaaatatagcgcgcaaactaggataaattatcgcgcgcggtgtcatctatgttactagatcgaccggc

atgcaagctgattatcaaaatctatttagaaatacacaatattttgttgcaggcttgctggagaatcgat

ctgctatcataaaaattacaaaaaaattttatttgcctcaattattttaggattggtattaaggacgctt

aaattatttgtcgggtcactacgcatcattgtgattgagaagatcagcgatacgaaatattcgtagtact

atcgataatttatttgaaaattcataagaaaagcaaacgttacatgaattgatgaaacaatacaaagaca

gataaagccacgcacatttaggatattggccgagattactgaatattgagtaagatcacggaatttctga

caggagcatgtcttcaattcagcccaaatggcagttgaaatactcaaaccgccccatatgcaggagcgga

tcattcattgtttgtttggttgcctttgccaacatgggagtccaaggttttaattaaactagtggtccaa

agaccagagggctattgagacttttcaacaaagggtaatatcgggaaacctcctcggattccattgccca

gctatctgtcacttcatcgaaaggacagtagaaaaggaagatggcttctacaaatgccatcattgcgata

aaggaaaggctatcgttcaagatgcctctaccgacagtggtcccaaagatggacccccacccacgaggaa

catcgtggaaaaagaagacgttccaaccacgtcttcaaagcaagtggattgatgtgatacatggtggagc

acgacactctcgtctactccaagaatatcaaagatacagtctcagaagaccagagggctattgagacttt

tcaacaaagggtaatatcgggaaacctcctcggattccattgcccagctatctgtcacttcatcgaaagg

acagtagaaaaggaagatggcttctacaaatgccatcattgcgataaaggaaaggctatcgttcaagatg

cctctaccgacagtggtcccaaagatggacccccacccacgaggaacatcgtggaaaaagaagacgttcc

aaccacgtcttcaaagcaagtggattgatgtgatatctccactgacgtaagggatgacgcacaatcccac

tatccttcgcaagacccttcctctatataaggaagttcatttcatttggagaggacacgctgaattcaca

acacaaatcagatttatagagagatttataaaaaaaaaaaaaacaatggagtggagctggatcttcttgt

tcttgctcagcggcactgcaggtgttcactccatggggtcttccaagaatgttatcaaggagttcatgag

gtttaaggttcgcatggaaggaacggtcaatgggcacgagtttgaaatagaaggcgaaggagaggggagg

ccatacgaaggccacaataccgtaaagctcaaggtaaccaaggggggacctttgccatttgcttgggata

ttttgtcaccacaatttcagtatggaagcaaggtcagttttacttcccttaattttctatgtactttcat

aattacttatgttattttcttcatgagttttaatgcaaattactatatggactctagtgaaaacgttcag

aatcctataaacatgactactgagacgaacttgagctttatgcccgggacaagtgaattcagtagttttg

atcatacacacgtttcatgtggtacttgagagttactaatttttgtcatcttcgtataagtagtaaaaga

tactacaagaatagtttagtagaaaatactagcggtaggtgaagatttgtcgctatgtactattattgtc

tagtaacttgagtaacaatttcgtggtctaaatatcaaataaaaatggatgagtggttcaccaaatctag

gcatcaaaactattaatgtcattgtctagatcttaggtgacaccacatttcgaatatttattggtaattg

agatgttaaagtaccaatatttgacttaataaactaaaagattttggctttatcaaatgtagacattgat

gacatatcgttgtcattatcttgagtatatacaagtcgatcaattaggtgaaagtttagtgtctcgtggt

tggtaaacgattaatacagtagtatattttatccaaagacaaaatccaaatcatttcaccagtatgaata

gtattattttatcttaaaagctaaaatcttaaaaaccaaggtagcacccacgttgagctagacgatcaaa

tcgatttctgctttgtccaatttaccaagctatttaaagccaaataattgaaatataggtaggtcgttat

attaggctaagatttatctcaaatgcttaactaaaggaataacaagggattctagttgtgtggttttata

agattggtccaatttcacttaagtttgtttattgtagaattttatatgtgaataatttgaattccgagct

cctcgagggggggcccggtacccagcttttgttccctttagtgagggttaatttcgagcttggcgtaatc

atggtcatagctgtttcctgtgtgaaattgttatccgctcacaattccacacaacatacgagccggaagc

ataaagtgtaaagcctggggtgcctaatgagtgagctaactcacattaattgcgttgcgctcactgcccg

ctttccagtcgggaaacctgtcgtgccagctgcattaatgaatcggccaacgcgcggggagaggcggttt

gcgtattgggcgctcttccgcttcctcgctcactgactcgctgcgctcggtcgttcggctgcggcgagcg

gtatcagctcactcaaaggcggtaatacggttatccacagaatcaggggataacgcaggaaagaacatgt

gagcaaaaggccagcaaaaggccaggaaccgtaaaaaggccgcgttgctggcgtttttccataggctccg

cccccctgacgagcatcacaaaaatcgacgctcaagtcagaggtggcgaaacccgacaggactataaaga

taccaggcgtttccccctggaagctccctcgtgcgctctcctgttccgaccctgccgcttaccggatacc

tgtccgcctttctcccttcgggaagcgtggcgctttctcatagctcacgctgtaggtatctcagttcggt

gtaggtcgttcgctccaagctgggctgtgtgcacgaaccccccgttcagcccgaccgctgcgccttatcc

ggtaactatcgtcttgagtccaacccggtaagacacgacttatcgccactggcagcagccactggtaaca

ggattagcagagcgaggtatgtaggcggtgctacagagttcttgaagtggtggcctaactacggctacac

tagaaggacagtatttggtatctgcgctctgctgaagccagttaccttcggaaaaagagttggtagctct

tgatccggcaaacaaaccaccgctggtagcggtggtttttttgtttgcaagcagcagattacgcgcagaa

aaaaaggatctcaagaagatcctttgatcttttctacggggtctgacgctcagtggaacgaaaactcacg

ttaagggattttggtcatgagattatcaaaaaggatcttcacctagatccttttaaattaaaaatgaagt

tttaaatcaatctaaagtatatatgagtaaacttggtctgacagttaccaatgcttaatcagtgaggcac

ctatctcagcgatctgtctatttcgttcatccatagttgcctgactccccgtcgtgtagataactacgat

acgggagggcttaccatctggccccagtgctgcaatgataccgcgagacccacgctcaccggctccagat

ttatcagcaataaaccagccagccggaagggccgagcgcagaagtggtcctgcaactttatccgcctcca

tccagtctattaattgttgccgggaagctagagtaagtagttcgccagttaatagtttgcgcaacgttgt

tgccattgctacaggcatcgtggtgtcacgctcgtcgtttggtatggcttcattcagctccggttcccaa

cgatcaaggcgagttacatgatcccccatgttgtgcaaaaaagcggttagctccttcggtcctccgatcg

ttgtcagaagtaagttggccgcagtgttatcactcatggttatggcagcactgcataattctcttactgt

catgccatccgtaagatgcttttctgtgactggtgagtactcaaccaagtcattctgagaatagtgtatg

cggcgaccgagttgctcttgcccggcgtcaatacgggataataccgcgccacatagcagaactttaaaag

tgctcatcattggaaaacgttcttcggggcgaaaactctcaaggatcttaccgctgttgagatccagttc

gatgtaacccactcgtgcacccaactgatcttcagcatcttttactttcaccagcgtttctgggtgagca

aaaacaggaaggcaaaatgccgcaaaaaagggaataagggcgacacggaaatgttgaatactcatactct

tcctttttcaatattattgaagcatttatcagggttattgtctcatgagcggatacatatttgaatgtat

ttagaaaaataaacaaataggggttccgcgcacatttccccgaaaagtgccacctgacgcgccctgtagc

ggcgcattaagcgcggcgggtgtggtggttacgcgcagcgtgaccgctacacttgccagcgccctagcgc

ccgctcctttcgctttcttcccttcctttctcgccacgttcgccggctttccccgtcaagctctaaatcg

ggggctccctttagggttccgatttagtgctttacggcacctcgaccccaaaaaacttgattagggtgat

ggttcacgtagtgggccatcgccctgatagacggtttttcgccctttgacgttggagtccacgttcttta

atagtggactcttgttccaaactggaacaacactcaaccctatctcggtctattcttttgatttataagg

gattttgccgatttcggcctattggttaaaaaatgagctgatttaacaaaaatttaacgcgaattttaac

aaaatattaacgcttacaatttccattcgccattcaggctgcgcaactgttgggaagggcgatcggtgcg

ggcctcttcgctattacgccagctggcgaaagggggatgtgctgcaaggcgattaagttgggtaacgcca

gggttttcccagtcacgacgttgtaaaacgacggccagtgaattgtaatacgactcactatagggcgaat

tggagctccaccgcggtggcggccgctctagaactagtggatcccccgggctgcaggaattcgatatcaa

gcttatcgataccgtcgacc

# >C) pBlueSKM_NHEJ_ZFN2_ZFN4

tcgagtttttcagcaagataagactcccgcccatagatctatgcccgggagaagtgactctctacatacc

ttgtggatttgtttacattgcctgatctttcctgtgatcgctgtcatgtttctttggaatgattgatgtt

tataaatggaaaaatctttgtgcaggtttcatcgactgtggccggctgggtgtggcggaccgctatcagg

acatagcgttggctacccgtgatattgctgaagagcttggcggcgaatgggctgaccgcttcctcgtgct

ttacggtatcgccgctcccgattcgcagcgcatcgccttctatcgccttcttgacgagttcttctgagcg

ggactctggggttcggactctagctagagtcaagcagatcgttcaaacatttggcaataaagtttcttaa

gattgaatcctgttgccggtcttgcgatgattatcatataatttctgttgaattacgttaagcatgtaat

aattaacatgtaatgcatgacgttatttatgagatgggtttttatgattagagtcccgcaattatacatt

taatacgcgatagaaaacaaaatatagcgcgcaaactaggataaattatcgcgcgcggtgtcatctatgt

tactagatcgaccggcatgcaagctgattatcaaaatctatttagaaatacacaatattttgttgcaggc

ttgctggagaatcgatctgctatcataaaaattacaaaaaaattttatttgcctcaattattttaggatt

ggtattaaggacgcttaaattatttgtcgggtcactacgcatcattgtgattgagaagatcagcgatacg

aaatattcgtagtactatcgataatttatttgaaaattcataagaaaagcaaacgttacatgaattgatg

aaacaatacaaagacagataaagccacgcacatttaggatattggccgagattactgaatattgagtaag

atcacggaatttctgacaggagcatgtcttcaattcagcccaaatggcagttgaaatactcaaaccgccc

catatgcaggagcggatcattcattgtttgtttggttgcctttgccaacatgggagtccaaggttttaat

taaactagtggtccaaagaccagagggctattgagacttttcaacaaagggtaatatcgggaaacctcct

cggattccattgcccagctatctgtcacttcatcgaaaggacagtagaaaaggaagatggcttctacaaa

tgccatcattgcgataaaggaaaggctatcgttcaagatgcctctaccgacagtggtcccaaagatggac

ccccacccacgaggaacatcgtggaaaaagaagacgttccaaccacgtcttcaaagcaagtggattgatg

tgatacatggtggagcacgacactctcgtctactccaagaatatcaaagatacagtctcagaagaccaga

gggctattgagacttttcaacaaagggtaatatcgggaaacctcctcggattccattgcccagctatctg

tcacttcatcgaaaggacagtagaaaaggaagatggcttctacaaatgccatcattgcgataaaggaaag

gctatcgttcaagatgcctctaccgacagtggtcccaaagatggacccccacccacgaggaacatcgtgg

aaaaagaagacgttccaaccacgtcttcaaagcaagtggattgatgtgatatctccactgacgtaaggga

tgacgcacaatcccactatccttcgcaagacccttcctctatataaggaagttcatttcatttggagagg

acacgctgaattcacaacacaaatcagatttatagagagatttataaaaaaaaaaaaaacaatggagtgg

agctggatcttcttgttcttgctcagcggcactgcaggtgttcactccatggggtcttccaagaatgtta

tcaaggagttcatgaggtttaaggttcgcatggaaggaacggtcaatgggcacgagtttgaaatagaagg

cgaaggagaggggaggccatacgaaggccacaataccgtaaagctcaaggtaaccaaggggggacctttg

ccatttgcttgggatattttgtcaccacaatttcagtatggaagcaaggtcagttttacttcccttaatt

ttctatgtactttcataattacttatgttattttcttcatgagttttaatgcaaattactatatggactc

tagtgaaaacgttcagaatcctataaacatgactactgagacgaacttggccttttgcagtttaagcttt

atgcccgggacaagtgatctttctagagcggccgccaccgcggtggagctccagcttttgttccctttag

tgagggttaatttcgagcttggcgtaatcatggtcatagctgtttcctgtgtgaaattgttatccgctca

caattccacacaacatacgagccggaagcataaagtgtaaagcctggggtgcctaatgagtgagctaact

cacattaattgcgttgcgctcactgcccgctttccagtcgggaaacctgtcgtgccagctgcattaatga

atcggccaacgcgcggggagaggcggtttgcgtattgggcgctcttccgcttcctcgctcactgactcgc

tgcgctcggtcgttcggctgcggcgagcggtatcagctcactcaaaggcggtaatacggttatccacaga

atcaggggataacgcaggaaagaacatgtgagcaaaaggccagcaaaaggccaggaaccgtaaaaaggcc

gcgttgctggcgtttttccataggctccgcccccctgacgagcatcacaaaaatcgacgctcaagtcaga

ggtggcgaaacccgacaggactataaagataccaggcgtttccccctggaagctccctcgtgcgctctcc

tgttccgaccctgccgcttaccggatacctgtccgcctttctcccttcgggaagcgtggcgctttctcat

agctcacgctgtaggtatctcagttcggtgtaggtcgttcgctccaagctgggctgtgtgcacgaacccc

ccgttcagcccgaccgctgcgccttatccggtaactatcgtcttgagtccaacccggtaagacacgactt

atcgccactggcagcagccactggtaacaggattagcagagcgaggtatgtaggcggtgctacagagttc

ttgaagtggtggcctaactacggctacactagaaggacagtatttggtatctgcgctctgctgaagccag

ttaccttcggaaaaagagttggtagctcttgatccggcaaacaaaccaccgctggtagcggtggtttttt

tgtttgcaagcagcagattacgcgcagaaaaaaaggatctcaagaagatcctttgatcttttctacgggg

tctgacgctcagtggaacgaaaactcacgttaagggattttggtcatgagattatcaaaaaggatcttca

cctagatccttttaaattaaaaatgaagttttaaatcaatctaaagtatatatgagtaaacttggtctga

cagttaccaatgcttaatcagtgaggcacctatctcagcgatctgtctatttcgttcatccatagttgcc

tgactccccgtcgtgtagataactacgatacgggagggcttaccatctggccccagtgctgcaatgatac

cgcgagacccacgctcaccggctccagatttatcagcaataaaccagccagccggaagggccgagcgcag

aagtggtcctgcaactttatccgcctccatccagtctattaattgttgccgggaagctagagtaagtagt

tcgccagttaatagtttgcgcaacgttgttgccattgctacaggcatcgtggtgtcacgctcgtcgtttg

gtatggcttcattcagctccggttcccaacgatcaaggcgagttacatgatcccccatgttgtgcaaaaa

agcggttagctccttcggtcctccgatcgttgtcagaagtaagttggccgcagtgttatcactcatggtt

atggcagcactgcataattctcttactgtcatgccatccgtaagatgcttttctgtgactggtgagtact

caaccaagtcattctgagaatagtgtatgcggcgaccgagttgctcttgcccggcgtcaatacgggataa

taccgcgccacatagcagaactttaaaagtgctcatcattggaaaacgttcttcggggcgaaaactctca

aggatcttaccgctgttgagatccagttcgatgtaacccactcgtgcacccaactgatcttcagcatctt

ttactttcaccagcgtttctgggtgagcaaaaacaggaaggcaaaatgccgcaaaaaagggaataagggc

gacacggaaatgttgaatactcatactcttcctttttcaatattattgaagcatttatcagggttattgt

ctcatgagcggatacatatttgaatgtatttagaaaaataaacaaataggggttccgcgcacatttcccc

gaaaagtgccacctgacgcgccctgtagcggcgcattaagcgcggcgggtgtggtggttacgcgcagcgt

gaccgctacacttgccagcgccctagcgcccgctcctttcgctttcttcccttcctttctcgccacgttc

gccggctttccccgtcaagctctaaatcgggggctccctttagggttccgatttagtgctttacggcacc

tcgaccccaaaaaacttgattagggtgatggttcacgtagtgggccatcgccctgatagacggtttttcg

ccctttgacgttggagtccacgttctttaatagtggactcttgttccaaactggaacaacactcaaccct

atctcggtctattcttttgatttataagggattttgccgatttcggcctattggttaaaaaatgagctga

tttaacaaaaatttaacgcgaattttaacaaaatattaacgcttacaatttccattcgccattcaggctg

cgcaactgttgggaagggcgatcggtgcgggcctcttcgctattacgccagctggcgaaagggggatgtg

ctgcaaggcgattaagttgggtaacgccagggttttcccagtcacgacgttgtaaaacgacggccagtga

attgtaatacgactcactatagggcgaattgggtaccgggccccccc
