# Supplementary material for: Targeted insertion of large DNA sequences by homology‐directed repair or non‐homologous end joining in engineered tobacco BY‐2 cells using designed zinc finger nucleases
Source: Plant Direct. 2019 Jul 19;3(7):e00153. doi: 10.1002/pld3.153 (PMC6639735; doi:10.1002/pld3.153)
Supplement: Supplementary file 7 [file PLD3-3-e00153-s007.docx]

**Table S1**: Sequences of primers used for analysis of TCLs and for analysis of HDR event candidates and NHEJ event candidates.

| **Primer name** | **Sequence 5’-3’** |
| --- | --- |
| **qPCR** |  |
| Nt_PAL_for  Nt_PAL_rev  Pnos_for  Pnos_rev  tGFP_for2  tGFP_rev2 | TACTATGACTTGATGTTGTGTGGTGACTGA  GAGCGGTCTAAATTCCGACCCTTATTTC  AACTGACAGAACCGCAACGTTG  ACTCTAATTGGATACCGAGGGG  CACACCACAATGGCTATGGAGT  ACATGGCTGAGAAGATACGGAGA |
| **junction PCR** |  |
| npt5_F5  nptII_3’UTR_rev2 | GACGTACTCGGATGGAAGCCGGTCTTGTC  CTTAAGAAACTTTATTGCCAAATGTTTGAACG |
| rfp5_F  rfp3 | GGTTCGCATGGAAGGAACGGTCAATGGGCACGAG  GCAAGGAACAGATGGTGGCGTCCCTC |
| dbbr_F2  RFP3_R2 | CTGTGGATAACCGTATTACCGCCTTTGAG  AGTGGAGAAGCTGTCCTCTAGGCAATG |
| **‘out-out’ PCR** |  |
| disR  disF | GTACATAGCGACAAATCTTCACCTACC  CCTGATTCTTCCGTGTCATCTCTG |
| **Sequencing** |  |
| rfp5  seq_LB_RV3  npt3_R1  npt5_F2 | GGAAGGAACGGTCAATGGGCACGAG  TGATAAAGCCAAAATCTTTTAG  CCACAGTCGATGAAACCTGCAC  TCGGATGGAAGCCGGTCTTGTC |
