# Supplementary material for: Targeted insertion of large DNA sequences by homology‐directed repair or non‐homologous end joining in engineered tobacco BY‐2 cells using designed zinc finger nucleases
Source: Plant Direct. 2019 Jul 19;3(7):e00153. doi: 10.1002/pld3.153 (PMC6639735; doi:10.1002/pld3.153)
Supplement: Supplementary file 8 [file PLD3-3-e00153-s008.docx]

Reply to remarks from reviewers for manuscript 2019-00278 “Targeted insertion of large DNA sequences by homology-directed repair or non-homologous end joining in engineered tobacco BY-2 cells using designed zinc finger nucleases”

We would like to thank the reviewers for their valuable input to our manuscript. We have addressed the reviewers’ remarks as follows:

Reviewer 1:

Figure 1 was simplified, i.e. the promoters are now plain colour. We felt that further simplification would lead to information being lost.

We have now used Agrobacterium throughout the manuscript.

Reviewer 2:

We have corrected the figure legend for Figure S5 – indeed, the figure refers to Figure 2b as it does now.
